# Directing Multicellular Organization by Varying the Aspect Ratio of Soft Hydrogel Microwells

**DOI:** 10.1002/advs.202104649

**Published:** 2022-04-17

**Authors:** Gayatri J. Pahapale, Jiaxiang Tao, Milos Nikolic, Sammy Gao, Giuliano Scarcelli, Sean X. Sun, Lewis H. Romer, David H. Gracias

**Affiliations:** ^1^ Department of Chemical and Biomolecular Engineering Johns Hopkins University Baltimore MD 21218 USA; ^2^ Department of Mechanical Engineering Johns Hopkins University Baltimore MD 21218 USA; ^3^ Maryland Biophysics Program Institute for Physical Science and Technology University of Maryland College Park MD 20742 USA; ^4^ Maryland Biophysics Program Institute for Physical Science and Technology and Fischell Department of Bioengineering University of Maryland College Park MD 20742 USA; ^5^ Department of Mechanical Engineering Cell Biology and Institute of NanoBioTechnology (INBT) Johns Hopkins University Baltimore MD 21218 USA; ^6^ Department of Cell Biology Anesthesiology and Critical Care Medicine Biomedical Engineering Pediatrics and Center for Cell Dynamics Johns Hopkins School of Medicine Baltimore MD 21205 USA; ^7^ Department of Chemical and Biomolecular Engineering Materials Science and Engineering Chemistry and Laboratory for Computational Sensing and Robotics (LCSR) Johns Hopkins University Baltimore MD 21218 USA; ^8^ Department of Oncology and Sidney Kimmel Comprehensive Cancer Center Johns Hopkins School of Medicine Baltimore MD 21205 USA

**Keywords:** curved geometry, hydrogels, protein patterning, self‐assembly, tissue engineering, tubulogenesis

## Abstract

Multicellular organization with precise spatial definition is essential to various biological processes, including morphogenesis, development, and healing in vascular and other tissues. Gradients and patterns of chemoattractants are well‐described guides of multicellular organization, but the influences of 3D geometry of soft hydrogels are less well defined. Here, the discovery of a new mode of endothelial cell self‐organization guided by combinatorial effects of stiffness and geometry, independent of protein or chemical patterning, is described. Endothelial cells in 2 kPa microwells are found to be ≈30 times more likely to migrate to the edge to organize in ring‐like patterns than in stiff 35 kPa microwells. This organization is independent of curvature and significantly more pronounced in 2 kPa microwells with aspect ratio (perimeter/depth) < 25. Physical factors of cells and substrates that drive this behavior are systematically investigated and a mathematical model that explains the organization by balancing the dynamic interaction between tangential cytoskeletal tension, cell–cell, and cell–substrate adhesion is presented. These findings demonstrate the importance of combinatorial effects of geometry and stiffness in complex cellular organization that can be leveraged to facilitate the engineering of bionics and integrated model organoid systems with customized nutrient vascular networks.

## Graphical abstract

A mode of cell organization in response to three‐dimensional geometry on soft substrates is presented. Cell organization is controlled by tuning the aspect ratio of the geometry, independent of chemical patterning and curvature. Cell and physical parameters regulating this cell organization are elucidated, used to guide cell organization in customized patterns, and investigate fundamental questions relevant to tissue development.

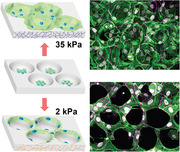

## 1 Introduction

Multicellular spatial organization drives morphogenesis, development, healing, and homeostasis,^[^ ^1^, ^2^, ^3^, ^4^, ^5^ ^]^ and its disruption has been implicated in the onset of diseases and the development of pathobiological processes as diverse as hypertension and malignancy.^[^ ^6^, ^7^, ^8^, ^9^, ^10^ ^]^ Multicellular organization is regulated by various environmental cues, including biochemicals such as signaling molecules and transcription or growth factors^[^ ^11^, ^12^, ^13^, ^14^ ^]^ and physical parameters like strain, shear, stiffness, and geometry.^[^ ^15^, ^16^, ^17^, ^18^, ^19^, ^20^, ^21^ ^]^ Researchers have reported that multicellular organization of vascular cells is controlled by the concentration and activities of biochemicals such as semaphorin 3E, platelet‐derived growth factor, and vascular endothelial growth factor, as well as physical factors including mechanical stretch and shear. Alteration of some of these factors may lead to pathologies like endothelial hyperplasia and abnormalities in capillary shape, diameter, and permeability.^[^ ^18^, ^22^, ^23^, ^24^, ^25^ ^]^ Similarly, developmental processes such as the formation of intestinal villi involve the sequential multicellular layered organization of intestinal cells in response to spatial gradients of differentiation and growth factors, leading to differential strain between layers and resulting in eventual folding to form the villi.^[^ ^26^, ^27^ ^]^

Recent advances in hydrogel synthesis and microscale patterning have led to clues concerning the interaction between physical cues such as stiffness and geometry in cell organization.^[^ ^20^, ^28^ ^]^ Matrix and substrate stiffness have been reported to affect the cell organization. For example, Paszek et al. report that epithelial cells aggregate to form lumenized structures resembling ducts on 150 and 400 Pa polyacrylamide hydrogels (moduli measured using parallel plate oscillatory shear rheometry). However, the organization was lost as stiffness increased, resulting in the formation of a monolayer on substrates with stiffness of 5 kPa or greater.^[^ ^8^ ^]^ Elsewhere, using either Matrigel‐collagen composite or polyacrylamide hydrogels, researchers have shown that endothelial cells typically undergo tubulogenesis on soft substrates with Young's moduli of 30–40 Pa (moduli measured by tensile testing)^[^ ^29^ ^]^ or 200–1000 Pa (moduli measured using parallel plate oscillatory shear rheometry)^[^ ^30^ ^]^ and spread to form a monolayer on substrates with stiffness >40 and ≥2500 Pa, respectively. The role of geometry in cell organization is most commonly probed by spatially confining cells using various adhesive (e.g., collagen, fibronectin) and nonadhesive (e.g., Pluronic^®^) 2D patterns.^[^ ^31^, ^32^, ^33^, ^34^, ^35^ ^]^ Recent studies have used 3D geometries to study cell alignment and migration, but these studies predominantly use substrates such as polydimethylsiloxane (PDMS) with nonphysiological stiffnesses of ≥1 MPa.^[^ ^36^, ^37^, ^38^ ^]^ Hence, the influence of geometry on multicellular organization in soft anatomically relevant 3D shapes is unclear.

**Figure 1:**
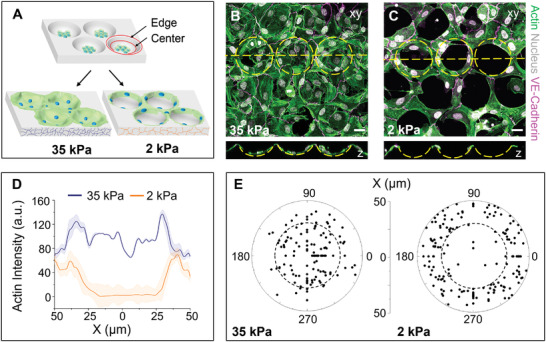
Multicellular organization is guided by geometry on soft 2 kPa hydrogels. A) Schematic depicting our findings of multicellular organization driven by geometry and stiffness in the absence of protein substrate patterns. We define two regions of equal projected area; edge (between the red circles) and center (inside the smaller red circle) to characterize the multicellular organization. B,C) Confocal images showing the top (*xy*) and side‐views (*z*) of actin (green), nucleus (gray), and VE‐cadherin (magenta) stained cells 24 h after seeding in hemispherical microwells. B) The images show uniform multicellular distribution all over the stiff 35 kPa hydrogel microwell area. C) In contrast, we observe a distinct ring‐like multicellular self‐organization around the edge of the soft 2 kPa hydrogel microwell. Yellow dashed lines indicate the positions of the microwells and the axis along which the side view is shown. Scale bar = 25 µm. D) Plot of actin intensity measured along the microwell diameter (*X*), in stiff 35 kPa and soft 2 kPa microwells. The plot depicts the average intensity ± SEM for 100 microwells over three independent experiments. E) Polar plot depicting the distribution of cells in the microwells quantified by measuring the distance of the nucleus center from the microwell center. The region inside the dotted circle indicates the center region of the microwell. This plot was generated from measurements using 200 cells from four independent experiments.

Here, we report a novel mode of endothelial cell self‐organization in the absence of any protein patterning, which occurs within microwells of soft hydrogels with a modulus of 2 kPa. Rather than a spatial pattern of adhesive and less adhesive regions dictating substrate affinity, the self‐organization we discovered is the consequence of the variation of two physical cues—namely, stiffness and microwell aspect ratio. We observe that more than 90% of the cells on soft 2 kPa hydrogels migrate toward and organize on the edge of the microwells, whereas cells on stiffer 35 kPa hydrogels uniformly spread throughout the microwells (**Figure** **1**A). These findings contrast with studies reported with fibroblasts, stem cells, and epithelial cells, in which cells on stiff substrates avoided microwells.^[^ ^36^, ^37^, ^38^ ^]^ We quantitatively characterize the multicellular self‐organization as a cell distribution ratio (*DR*) that is defined as the ratio of the number of nuclei on microwell edge (as the numerator) to the number of nuclei in the center (as the denominator). We report a strong correlation between the organization of cell populations and the microwell aspect ratio (*ε*), defined as the ratio of microwell perimeter to depth on soft hydrogels. We investigate this phenomenon by measuring *DR* in the context of different levels of cell contractility (cell tension), cell density, and cell adhesion and test our findings by implementing a force balance‐based mathematical model. Apart from affecting multicellular organization in periodic microwells, we further show that geometric adjustments in soft hydrogels can be used to self‐organize cells in various CAD‐designed microwell shapes, such as the letters of the word “CELL.” We believe that our discovery substantially advances understanding of the complex role of geometry and stiffness on multicellular organization. We anticipate that this discovery will guide the design of microenvironments that direct multicellular organization for complex devices, mimetic tissues, and organoid designs in the absence of protein patterning.

## 2 Results

### 2.1 Endothelial Cells Self‐Organize into Ring‐Like Patterns on Soft Hydrogel Microwells in the Absence of Protein Patterns

We used photolithography and molding to pattern hydrogels of Young's moduli 2 and 35 kPa with microwells 80 µm in diameter and 25 ± 2 µm in depth (Figure S2, Supporting Information). We chose these dimensions to mimic geometries found in anatomical structures, including arteries, mammary acini, intestinal crypts, alveoli, and epidermis.^[^ ^39^ ^]^ We arranged the microwells periodically in a hexagonal array where the spacing between two adjacent microwells is at least 5 µm. We determined the mechanical properties of the hydrogels using oscillatory shear rheometry and found that the hydrogel mechanical properties do not change over time (measured over 20 min) or under strain (measured between 0% and 10%) (Figure S3A,B, Supporting Information). We also used atomic force microscopy (AFM) to measure the local mechanical properties of the micropatterned hydrogel (Figure S3C, Supporting Information). The moduli obtained from AFM measurements are lower than those from the rheological measurements. However, the relative difference between the soft and stiff hydrogel moduli is of a similar magnitude (the ratio of soft‐to‐stiff moduli is 0.04 from AFM measurements and 0.05 from rheology measurements).

On seeding human umbilical vein endothelial cells (HUVECs) in the hydrogel microwells, we observed a striking difference in cell organization based on hydrogel stiffness. Cells in stiff 35 kPa microwells organized into a uniform monolayer in and around the microwells (Figure 1B), whereas cells in soft 2 kPa microwells self‐organized exclusively (>90%) on the periphery of microwells in the shape of rings (Figure 1C). We have used gelatin hydrogels for the majority of our experiments and verified this self‐organization with polyacrylamide hydrogels (Figure S4, Supporting Information).

Using live‐cell imaging, we observed that HUVECs in soft 2 kPa microwells always started at the center of the microwells and then gradually moved to the edges of the microwell (Movie S1, Supporting Information). After 8–10 h of seeding, most of the cells in soft 2 kPa microwells were on the microwell periphery. While we observed that cells continued to move along the microwell edge for the remaining observation duration (cells are observed for a total of 24 h), we did not observe significant changes in the cellular organization beyond this time point and refer to it as the steady‐state.

We categorized the cells in the microwells into two regions of equal projected area: microwell edge and microwell center (Figure 1A). We observed that in many cases, especially on the stiff hydrogels, the entire cell body was not localized in a single microwell, but the nuclei were either entirely in the center or on the edge. Thus, we approximated the cell's location in microwells by the location of the nucleus, which has been used as a position marker by multiple studies.^[^ ^38^, ^40^, ^41^ ^]^ We defined the cell *DR* as the ratio of nuclei on edge to center and quantified it by counting the number of cells in the respective microwell regions (nuclear position is measured from the microwell center, detailed in the Experimental Section). We observed that the steady‐state *DR* for cells in soft 2 kPa microwells (*DR* ≈ 15) was ≈30 times that for cells in stiff 35 kPa microwells (*DR* ≈ 0.5) (Figure 1E). We also measured the average actin fluorescent intensity in the microwells by using phalloidin‐labeled actin. We found minimal (0 ± 10 a.u. (arbitrary units)) actin in the central region with most (≈70 ± 10 a.u.) of the actin accumulating on the edges of soft 2 kPa microwells (Figure 1D and Figure S5, Supporting Information). In contrast, for stiff 35 kPa microwells, the actin signal is higher and observed all over the microwell (between 80 and 120 a.u.). Thus, the actin distribution is in agreement with *DR*. The drop in intensity observed at the 35 kPa microwell center results from the nuclear clustering at the microwell center.

In addition to the 2 and 35 kPa, we also seeded cells on 8 kPa microwells to examine the effect of a medium stiffness substrate on cell organization. We observed that cells in 8 kPa microwells demonstrate behavior intermediate to cells in 2 and 35 kPa microwells with a *DR* of 3.8 (Figure S6, Supporting Information). Thus, to meaningfully examine the effect of geometry, we used only the 2 and 35 kPa microwells for further studies.

### 2.2 The Topography and Mechanical Properties of the Hydrogel Microwells are Uniform

**Figure 2:**
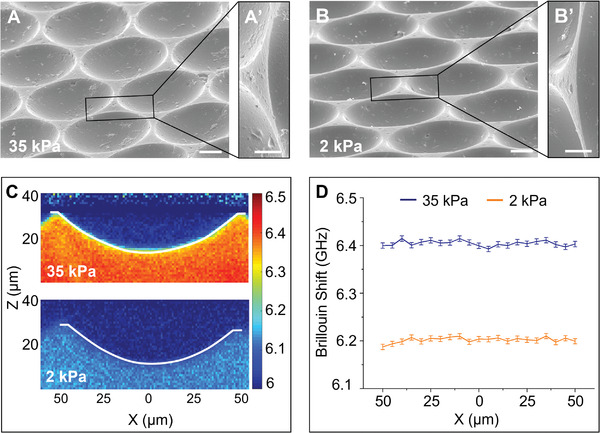
Microwells are topographically and mechanically homogenous. A,B) Tilted SEM micrographs of stiff 35 kPa and soft 2 kPa microwells with magnified sections (A’, B’) to focus on the edge of the microwells showing that surface topography of the microwells is homogenous and similar around the edges. Scale bar = 10 µm for (A, B) and 5 µm for (A’, B’). C) Representative Brillouin images of stiff 35 kPa and soft 2 kPa microwell with the white curved line indicating the boundary between PBS and the hydrogel. Heat map scale indicates Brillouin shift in GHz. D) Brillouin shifts on the microwell surface along the diameter (*X*) just below the hydrogel–liquid interface. Data presented are mean ± SD from three samples.

We characterized the topography and mechanical homogeneity of the gelatin microwells using scanning electron microscopy (SEM), optical microscopy, and Brillouin microscopy. SEM images taken at a tilt angle of 60° show that the surfaces of the soft 2 kPa and stiff 35 kPa microwells are uniformly flat and that there is no topographical irregularity (**Figure** **2**A,B). We also observed that the self‐organization in soft 2 kPa microwells occurred consistently as verified over large microwell patterned areas (1 mm × 1 mm) using fluorescence microscopy, thus confirming that the microwell patterns are spatially uniform (Figure S7, Supporting Information). Next, we examined the pore structure of the hydrogels and observed heterogeneity in porosity which is expected within hydrogels of different stiffnesses, with pores in low molecular weight gelatin (2 kPa) being generally smaller compared to higher molecular weight (35 kPa) gelatin hydrogels (Figure S8, Supporting Information). However, we note that cell organization similar to that in gelatin microwells is observed in polyacrylamide microwells of the same stiffness (Figure S4, Supporting Information), which typically have pores smaller in size than those in gelatin hydrogels.^[^ ^42^, ^43^ ^]^

To ensure that mechanical heterogeneities were not driving cell migration to the microwell edge via durotaxis,^[^ ^44^, ^45^ ^]^ we used contact‐independent Brillouin microscopy to map the local mechanical properties of the hydrogels with optical resolution in a hydrated state.^[^ ^46^, ^47^ ^]^ The Brillouin frequency shift is sensitive to changes in material elastic modulus and has previously been used to characterize several types of soft and biological materials,^[^ ^48^ ^]^ including gelatin‐based hydrogels.^[^ ^49^ ^]^ We quantified the Brillouin frequency shift of the hydrogel cross‐sections and found it constant within 0.08% relative error on all microwell surfaces (Figure 2C,D and Figure S9A,B, Supporting Information). We compared the values of Brillouin shift in the microwells and the flat part between the microwells with that of flat hydrogels made from the same gelatin type (Figure S9C, Supporting Information) and found that they were nearly identical, indicating that patterning of the hydrogels does not alter the mechanical properties of the hydrogel substrates. Furthermore, our observation that the Brillouin shift is homogenous across the microwell surface confirms the mechanical uniformity of the hydrogel microwells.

### 2.3 Self‐Organization Occurs in Soft 2 kPa Microwells of Different Shapes

We studied multicellular self‐organization in hemispherical, cylindrical, cubic, and pyramidal microwells. In all cases, we observed self‐organization at the edges of only the soft 2 kPa microwells without significant bias in *DR* for any of the studied shapes (Figure S10, Supporting Information). This observation indicated that the circular shape of the microwell did not specifically induce the observed multicellular organization. Also, the fact that cells self‐organized on the top edge of both hemispheres (one circular edge on the tops of the microwells) and cylinders (two circular edges, one at the top and one at the bottom of each microwell) indicated that neither contact guidance nor curvature is driving the observed self‐organization.^[^ ^38^, ^50^ ^]^ We used hemispherical microwells for all our further studies following these observations.

### 2.4 Self‐Organization Depends on the Microwell Aspect Ratio

**Figure 3:**
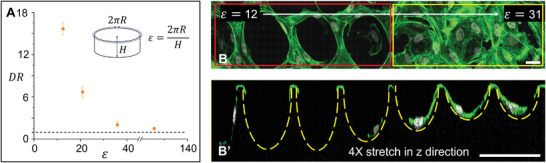
Multicellular organization in 2 kPa microwells depends on microwell aspect ratio. A) Plot depicting the relation between multicellular organization as quantified by *DR* and the aspect ratio *ε*, for the soft 2 kPa microwell. Data presented are mean ± SD and are quantified from 100 cells for each aspect ratio from three experiments. B,B’) Confocal images showing B) the top (*xy*) and B’) side‐views (*z*) of cells stained for actin (green) and nucleus (gray) in soft 2 kPa microwells with decreasing depth (increasing *ε*) from left to right. Red box depicts microwells with depth > 10 µm (*ε* < 25) and yellow box depicts microwells with depth < 10 µm (*ε* > 25). The image in panel (B’) is stretched by a factor of four in the *z*‐direction, to clearly visualize the shallower microwells. Yellow dashed arcs are included to depict the microwell shape in the *z* plane. Scale bar = 25 µm for the *x*‐direction in (B) and *z*‐direction in (B’).

In our preliminary experiments, we observed that HUVECs formed a monolayer on flat soft 2 kPa hydrogels (Figure S11, Supporting Information). This observation indicated that microwell depth is a driving factor in the self‐organization of cells into ring‐like structures. We, therefore, investigated the influence of microwell geometry on cell organization in detail by varying the microwell aspect ratio (*ε*) of soft 2 kPa microwells. We systematically varied the microwell depth from 25 to 0.5 µm while keeping the perimeter constant at 250 µm. We observed that for microwells deeper than 10 µm (*ε* < 25), cells migrated away from the center to self‐organize around the edge with a *DR* > 5 (**Figure** **3**A,B red box and Figure S12A, Supporting Information). For microwells less than 10 µm deep (*ε* > 25), more cells tend to stay in the center region. The distribution of cells between the microwell edge and center approaches equality (*DR*→1) for microwells with depth less than 2 µm (Figure 3A,B yellow box and Figure S12B, Supporting Information). A similar trend was observed for soft 2 kPa microwells with 470 µm perimeter (Figure S13, Supporting Information). These observations indicated that the microwell aspect ratio is critical in determining cell organization.

### 2.5 Multicellular Self‐Organization Occurs at an Optimal Cell Density

We observed that the cell movement toward the edges of soft 2 kPa microwells strongly depended upon the cell seeding density. At high seeding densities (>5 × 10^4^ cells cm^−2^), cells occupied the entire microwell area in both stiff 35 kPa and soft 2 kPa microwells and did not organize in ring‐like structures. However, when seeded at lower densities (≈6.5 × 10^3^ cells cm^−2^), we found that the cells in the stiff 35 kPa, as well as soft 2 kPa microwells, kept moving in the microwell center (Figure S14A,B and Movie S2, Supporting Information). Interestingly, when independently moving and noncontiguous cells in soft 2 kPa microwells encountered one another (13 h timestamp, Movie S2, Supporting Information), they tended to move toward the microwell edge. For cells in soft 2 kPa microwells at a very low density, the *DR* was less than one and decreased as the microwell depth increased (or as *ε* decreased), suggesting that cell migration toward the microwell edge may be enhanced by the presence of neighboring cells (Figure S15, Supporting Information). We found that the optimal seeding density that allowed the cells to self‐organize on the soft 2 kPa microwell edge was between 2‐3 × 10^4^ cells cm^−2^. At this optimal density, cells seeded in soft 2 kPa microwells moved toward and along the edges and exhibited greater translational motion than cells in stiff 35 kPa microwells (Figure S14C,D, Supporting Information). These observations suggested that cell–cell interactions play a role in cell self‐organization at the microwell edges in 2 kPa microwells.

### 2.6 Delineating the Role of Substrate Stiffness on Multicellular Self‐Organization by Regulating Cell Contractility

Multicellular self‐organization into ring‐like structures only occurred in soft 2 kPa microwells and not stiff 35 kPa microwells. This observation indicates that substrate stiffness is a regulating factor in guiding the observed self‐organization. It is known that substrate stiffness affects cell size, cytoskeletal contractility, and cell adhesions.^[^ ^51^, ^52^, ^53^, ^54^ ^]^ Since stiffer substrates lead to a larger spreading area which may lead to the filling of the microwells, we first investigated the cell size in the microwells. We measured the cell area by projecting the confocal z‐stack on a plane and found that the sizes of the cells in 35 kPa and that of the cells in 2 kPa microwells were similar (Figure S16A, Supporting Information). We also examined cell proliferation by counting cell division events per microwell using the live cell time‐lapse movies and found that cell proliferation was ≈0.5 cell divisions/microwell/20 h in both 35 and 2 kPa microwells (Figure S16B and Table S2, Supporting Information). We thus hypothesized that the difference in cell behavior could partly be due to the difference in cytoskeletal contractility on the two hydrogels. Previous studies have established that substrate stiffness can be correlated to cell contractility and that cell contractility increases as substrate stiffness increases.^[^ ^51^, ^52^, ^53^ ^]^ We tested this hypothesis by altering the cell contractility using pharmacological agents.

Specifically, we suppressed Rho‐activated, myosin II‐dependent contractility of the microfilament cytoskeleton in cells by inhibiting ROCK with Y27632 (30 × 10^−6^ m). In parallel experiments , we inhibited myosin dephosphorylation and inactivation using the phosphatase inhibitor Calyculin A (0.1 × 10^−9^ m) to increase the contractile state of the microfilament cytoskeleton. We observed that ROCK inhibition in cells seeded to stiff 35 kPa microwells led to self‐organization on the edge of microwells similar to that seen in untreated cells on soft 2 kPa microwells (Figures S17A,E and S18, Supporting Information). Interestingly, reciprocal conditions whereby Calyculin A treated cells seeded in soft 2 kPa microwells yielded the accumulation of these cells in the microwell centers in a manner that recapitulated the behavior of untreated cells on stiff 35 kPa microwells (Figures S17D,F and S18 Supporting Information). ROCK inhibitor‐treated cells in 2 kPa microwells continued to organize on the microwell edge (Figures S17B,E and S18, Supporting Information) and Calyculin A‐treated cells in 35 kPa microwells continued to spread inside the microwell (Figures S17C,F and S18, Supporting Information). These results support the hypothesis that cytoskeletal contractility (as dictated by the substrate stiffness) is a significant contributor in directing cell self‐organization and that stiffer substrates inhibit the geometry‐enhanced cell motility observed in microwells comprised of a softer substrate.

### 2.7 Influence of Cell–Cell and Cell–Substrate Interaction on Multicellular Self‐Organization

Cell–cell and cell–substrate interactions are strongly influenced by stiffness and cell density. Prior reports show that increased substrate stiffness promotes robust cell–substrate interaction through focal adhesions (FAs) due to force loading and increased protein clustering,^[^ ^54^, ^55^ ^]^ whereas increased substrate stiffness destabilizes VE‐cadherin‐mediated cell–cell junctions and weakens cell–cell interactions.^[^ ^56^, ^57^ ^]^ We quantitatively investigated the effect of the regulating parameters, microwell stiffness, size, and cell density on cellular interactions and their impact on our observed multicellular self‐organization by examining the expression and localization of cell–substrate and cell–cell adhesion molecules.

We began by studying paxillin in focal adhesions at 3 and 24 h after cell seeding to quantify the 3D FA surface area and volume. We grouped the FA into two categories—microwell edge or microwell center. FAs produced by cells that extended from the microwell edge to the microwell center were categorized in the appropriate group based upon the FA location. We also studied VE‐cadherin at EC cell–cell junctions after 24 h of seeding in soft 2 kPa and stiff 35 kPa microwells.

**Figure 4:**
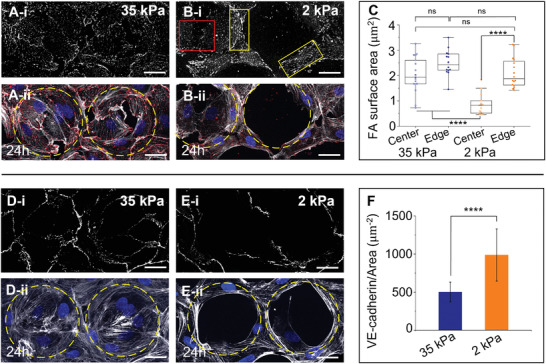
Cell–substrate and cell–cell interactions at the center and edge of soft and stiff microwells. A‐i,B‐i) Confocal images showing the top view of cells immunolabeled for paxillin (gray) 24 h after seeding in stiff 35 kPa and soft 2 kPa microwells, respectively and A‐ii,B‐ii) the corresponding composite top view of cells labeled for paxillin (red), actin (gray), and nucleus (blue). Yellow box shows the streak‐like FA on edge, and the red box shows the dot‐like FA inside soft 2 kPa microwells. Yellow dashed circles indicate the microwell position. Scale bar = 25 µm. C) Box‐whisker plot depicting the FA size for cells at the center and edge of stiff 35 kPa and soft 2 kPa microwells. Data presented are for ≥ 15 cells from three independent experiments. ****p values < 0.0001, ***p value < 0.001, and ns > 0.05 calculated by one‐way ANOVA followed by Tukey's mean comparison. D‐i,E‐i) Confocal images showing the top view of cells labeled for VE‐cadherin (gray) 24 h after cell seeding in stiff 35 kPa and soft 2 kPa microwells, respectively, and D‐ii,E‐ii) the corresponding top view for cell stained for actin (gray) and nucleus (blue). Yellow dashed circles indicate the microwell position. Scale bar = 25 µm. F) Plot depicting average VE‐cadherin intensity per unit junction area. Data presented are mean ± SD for >10 sample areas pooled from three independent experiments. ****p value < 0.0001 calculated by Student's *t*‐test.

We observed that cells in stiff 35 kPa microwells generally established larger, streak‐like FAs. At 3 h, FAs produced at the edges of the 35 kPa microwells were larger than those in the microwell center (Figure S19A‐i,C, Supporting Information), and this difference was resolved by the end of 24 h (**Figure** **4**A‐i,C). In contrast, at both 3 and 24 h time points, FAs in HUVECs at the center of the soft 2 kPa microwells were smaller and predominantly punctate structures (Figure 4B‐i, red box and Figure S19B‐i, Supporting Information), while those at the edge were streak‐like FAs and ≈2.5 times larger than those in the center (Figure 4B‐i, yellow box, C and Figure S19B‐i,C, Supporting Information). Moreover, FA size on the edges of the soft 2 kPa microwells was comparable to the FA size measured in stiff 35 kPa microwells at 24 h (Figure 4C and Figure S20A,B, Supporting Information). A similar distribution of FA was observed in cells seeded at low and very high density in the soft 2 kPa microwells (Figure S20C,D‐i, Supporting Information).

Thus, the limited translational motion of the cells in the 35 kPa microwells may be attributed in part to the presence of large FAs made by cells inside microwells (Figure S14C, Supporting Information). In contrast, cells formed much smaller FAs inside soft 2 kPa microwells, facilitating their motility out of the microwell center and toward the edges, where they established large FAs and stabilized their position (Figure S14D, Supporting Information). Since inhibition of ROCK reduces the formation of FAs due to reduced stress fiber formation,^[^ ^58^ ^]^ Y27632 may cause cells in 35 kPa microwells to mimic the behavior of cells in 2 kPa microwell centers and migrate to the edge.

VE‐cadherin localized along the majority of each cell's perimeter in stiff 35 kPa microwells, as HUVECs there are tightly contiguous to neighboring cells. In contrast, cells in soft 2 kPa microwells only exhibited cell–cell contacts along ≈40–50% of their perimeter (Figure 4D,E). We quantified the VE‐cadherin intensity and normalized these values per unit of junctional area and found that the normalized VE‐cadherin intensity for cells in soft 2 kPa microwells was approximately twice that of cells in stiff 35 kPa microwells (Figure 4F). Thus, in addition to forming larger FA, cells at the edges of the soft 2 kPa microwells also formed more cell–cell junctions heavily populated by VE‐cadherin. This finding also explains the behavior of single cells inside the 2 kPa microwell, where they cannot migrate to the edge (Figure S14B, Supporting Information).

Thus, it is evident from our experiments that the multicellular self‐organization that we observe involves an interplay between cell–cell and cell–substrate interactions and that this dynamic is influenced by the three parameters noted above: substrate stiffness, microwell aspect ratio, and cell plating density.

### 2.8 Balance between Cell Tension, Adhesion, and Cohesion Can Explain the Observed Cell Organization

**Figure 5:**
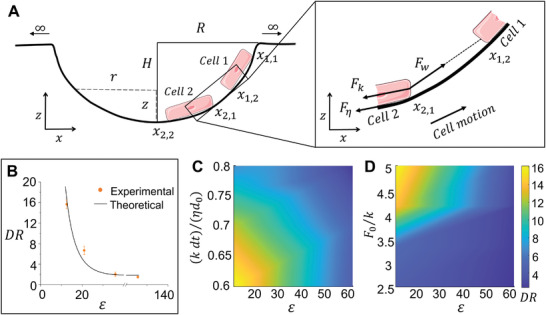
Force balance model predicts the observed pattern of multicellular organization. A) Schematic depicting the force balance model parameters. The model analyzes the cell tension (*F_k_*), cohesion (*F_w_*), and adhesion (*F_*η*_*) forces, assumed to be concentrated at the end nodes of the cells, to predict the observed multicellular organization. B) Plot depicting the experimental observations and theoretical predictions for the relationship between cell arrangement as characterized by *DR* and microwell aspect ratio (*ε*). C,D) Plot depicting the *DR* for various aspect ratios (*ε*) as a function of C) cytoskeletal tension and D) cell–cell interaction (cell density).

We developed a mathematical model integrating our findings to theoretically explain the observed multicellular self‐organization (see Supporting Information for full description). We modeled the force‐balance relationships and active force generation by cells to simulate the dynamics of 2 (**Figure** **5**A) and 3–4 cells (Figure S21A,E‐J Supporting Information) moving along the microwell wall with a depth *H* and radius *R*. We use a 2D model in which the cell motion is projected on the *x*–*z* plane. Such a model can be translated to 3D and allows predicting the cell behavior along the appropriate cross‐section of any microwell shape(Note S1, Supporting Information). We simplified the cell shape with a line segment and assumed that all the forces that affect cellular shape and dynamics collectively act at the cell's endpoints (nodes). The direction of cytoskeleton tension and cell–cell interactionforce is along the linear direction of two affected nodes (Figure 5A and Note S1, Supporting Information). Therefore, more complicated assumptions regarding cell shape, such as curved or 3D features, may not be necessary, and more complex cell shape characteristics would not change the results. We can write the force‐balance equation at *i*th endpoint of *j*th cell, *x*_*j*,*i*_, as

(1) $$\eta \;{\dot{x}}_{j,i}=-\frac{d}{dx_{j,i}}\left[\frac{k}{2d_{0}}{\left(d_{j}-d_{0}\right)}^{2}\right]+Af_{j,i}+F_{0}\sum_{m,k}w\left(x_{j,i},x_{m,k}\right)$$

where the left‐hand of the equation is the cell–substrate interaction (adhesion) described as the sliding frictional force (*F_*η*_*) between the cell and the substrate with coefficient *η*. The right‐hand side describes cell active forces comprising volume (length) regulation by cellular cytoskeletal forces (*F_k_*) and active protrusion/contraction. The last term on the right‐hand side of Equation (1) describes the cell–cell interaction (cohesion) (*F_w_*).

We describe the cell cytoskeletal tension mechanics using a spring potential with the stiffness of *k*, which is directly related to the cell's cytoskeletal stiffness, equilibrium length (*d*_0_), and current length (*d_j_*). The active protrusions and contractions, ***f*** *_j,i_*, generated by each cell is modeled by a random Gaussian noise term with zero mean, and scaled by a constant *A*. We assumed that the interaction force between the *i*th node of the *j*th cell and the *k*th node of the *m*th cell, ***w*** (***x*** *_j,i_*, ***x*** *_m,k_*), mediated by VE‐cadherin, follows a van der Waals‐like potential that contains short‐range repulsion and long‐range attraction (Figure S21B, Supporting Information). The cell–cell interaction strength is related to cell seeding density and the overall expression of VE‐cadherin and is described by a scaling constant *F*_0_ in our model. This interaction force is along the direction connecting the two cell endpoints: ***x*** *_m,k –_* ***x*** *_j,i_*.

The force equation of motion can explicitly compute the cell velocity and indicates that any unbalanced force between tension, cell–cell interaction, and random protrusions at each node must be compensated by the local motion along the microwell (which generates frictional force). Since the cell active forces are along the cellular orientation, the model suggests that the force is most likely to balance when cellular orientation is aligned with the local tangential direction. Specifically, the cell orientation is roughly along the local tangential direction toward the top edge in deeper microwells (*ε* < 25) and progressively shifts toward the center as the microwells become shallower (*ε* > 25) (Figure S21C, Supporting Information). Thus, the cells are more likely to stay near the top edge in deeper microwells while toward the center in shallower microwells (Figure 5B), explaining the observed migration of cells to the edges of microwells.

Our model also explains the cellular organization in the microwell when cellular active forces, cell–cell, and cell–substrate interactions are varied to account for changes in microwell stiffness and cell density. Usually, higher cell stiffness, *k*, or lower cell–cell interaction strength, *F*_0_, requires a smaller tangential component of overall cellular tension for the force balance to be equal. Therefore, when cells are placed in stiff 35 kPa microwells, the resulting higher cytoskeletal stiffness and lower VE‐cadherin activities than cells seeded in soft 2 kPa microwells tend to move the cells more toward the center to compensate for the extra increase in cell tension even in deeper microwells (Figure 5C and Figure S21E,G, Supporting Information). On the other hand, when cells are seeded at a higher density in 2 kPa microwells, which increases the cell–cell interaction strength, *F*_0_, a higher tangential component of cell tension is needed to balance the increased interaction leading to cell migration toward the edge of the deeper microwell. Thus, when cells in soft microwells contact a second cell, the cells move more toward the top edge in microwells with a favorable aspect ratio and greater depth (Figure 5D and Figure S21F,H, Supporting Information).

## 3 Discussion

The physiological landscape features niches with heterogenous mechanical and geometrical properties. These properties elicit diverse cell–extracellular matrix (ECM) interactions, such as the cycling of cell adhesions and cytoskeletal stiffness changes that influence cell aggregation and tissue organization.^[^ ^59^, ^60^ ^]^ Our ability to create 3D micropatterns in hydrogels as soft as 2 kPa allows us to recreate anatomically relevant features that drive striking differences in self‐organization using materials with physiologic stiffness. Our approach contrasts with most prior studies that utilize PDMS, which has a substantially higher Young's modulus than materials that characterize most biological tissues.^[^ ^37^, ^38^, ^61^, ^62^ ^]^

We have described a new mode of multicellular organization in response to physical cues that regulate cell migration and tissue morphogenesis. Specifically, we show that the soft microwell aspect ratio at cellular length scales can drastically influence the self‐organization of endothelial cells (ECs). We show that cells demonstrate a predilection to stay in locations where they can form stable cell–substrate adhesion and strong cell–cell interactions. We explain this finding mathematically by considering changes in the balance between cytoskeletal tension and extracellular forces that include cell–cell and cell–substrate adhesion. This force balance can be adjusted by substrate stiffness, the aspect ratio of microenvironmental geometry, and cell density.

Studies have demonstrated that the physical properties of ECM and cell density both affect EC stiffness and EC interactions with substrate and with other cells. Yeung et al. demonstrated that ECs on 28.6 kPa polyacrylamide substrates have higher cytoskeletal stiffness than those soft 180 Pa substrates.^[^ ^51^ ^]^ Stroka and Aranda‐Espinoza reported the effect of cell density on cell stiffness where individual ECs were stiffer than those in a monolayer, and decreasing cell–cell adhesion in a monolayer increased the cell stiffness.^[^ ^63^ ^]^ Further, Deronne et al. show that ECs on stiff substrates (≈80 Pa) make larger and stronger FAs than those on soft substrates (≈30 Pa) and do not undergo tubulogenesis.^[^ ^29^ ^]^ Finally, Califano and Reinhart‐King report that ECs moving in pairs on stiffer substrates (≈10 kPa) generate a higher traction force (a measure of cell–substrate interaction) than on soft substrates (1 kPa), and traction forces generated by independently moving ECs are lower than those generated by cell pairs in either environments.^[^ ^64^ ^]^

Our discovery demonstrates that cells in 3D geometries on soft substrates integrate these responses to extracellular cues to self‐organize in unique patterns. An intriguing finding in our study is that the geometry of the microwell—specifically the aspect ratio (*ε*) impacts the magnitude of cytoskeletal tension that balances the extracellular interactions and hence multicellular organization. This impact of the microwell aspect ratio is due to the balance of cell‐intrinsic and extracellular forces in the tangential direction at steady‐state (Figure 5A). Cells at optimal density (2–3 × 10^4^ cells cm^−2^) in soft microwells have lower overall cytoskeletal tension, lower cell–substrate interaction at the centers than on the edges of the microwells, and higher cell–cell interaction. This imbalance drives the cell motion to the edge, where the larger tangential component of cell tension and higher traction (friction) generated due to cell–substrate interaction balance the cell–cell interaction. In contrast, cell–cell interactions in stiff microwells can be balanced at any location in the microwell due to the elevated levels of cell tension and cell–substrate interaction. We demonstrate that perturbing any of these three forces drastically changes the cell organization. Increasing cell tension in soft microwells by either increasing cell contractility or reducing cell density results in the loss of self‐organization in soft microwells. The opposite is true for cells in stiff microwells, where decreasing cell tension leads to self‐organization on the edges of the microwells (Figure 5 and Figure S17, Supporting Information).

Such perturbations in vivo can lead to lethal pathologies. For example, ECs organize into shorter or severely tortuous blood vessels in pathological conditions like hypertension, diabetes, and other vascular conditions with altered vascular wall mechanics.^[^ ^65^, ^66^, ^67^ ^]^ The inability of cells to organize into healthy blood vessels is partly attributed to ECM stiffening. Also, in the case of tumor blood vessels, elevated cell proliferation has been reported to affect cell organization resulting in randomly structured and leaky blood vessels that lack the hierarchical structure of a normal vasculature.^[^ ^68^ ^]^ Our experimental and theoretical insights into the mechanics that modulate morphogenesis may be harnessed to design in vitro microenvironments to study diverse cellular populations and their organization during normal development, aging, and disease pathogenesis.

From a technological perspective, our discovery's key significance is that cell organization can be directed by defining the aspect ratio of the 3D geometry on soft hydrogels without the need for patterning cell adhesive and repellent regions or modulating substrate curvature. It is noteworthy that most prior literature utilizes patterns of chemoattractants or mechanical properties on flat substrates to manipulate cell organization.^[^ ^33^, ^44^ ^]^ In contrast, we guide multicellular organization without protein patterning. Thus, our approach to cell patterning offers a unique advantage to study cell organization in 3D and can provide avenues for co‐culture systems to understand the interaction between endothelial cells and other cell types.

**Figure 6:**
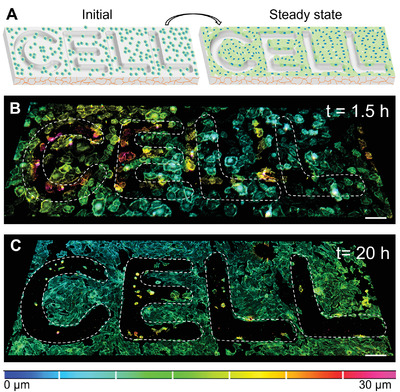
Directing multicellular organization in a predefined customizable pattern driven only by substrate geometry. A) Schematic representing initial and steady‐state cell distribution on soft 2 kPa hydrogels patterned with the word CELL. B) Depth‐coded confocal 3D stack of LifeAct‐GFP‐expressing HUVECs on soft 2 kPa hydrogel patterns 1.5 h (initial) after seeding. See Figure S22B (Supporting Information) for the brightfield image which shows all the cells. C) Depth coded confocal 3D stack of the same LifeAct‐GFP‐expressing HUVECs in (B) which were fixed and stained for actin to visualize all the cells on soft 2 kPa hydrogel patterns 20 h after seeding (*t* > steady‐state). Scale bar = 100 µm.

Our biofabrication approach utilizes photolithography and replica molding and is amenable to creating customizable shapes of hydrogel microwells. Thus, we can direct multicellular self‐organization without protein patterning in various CAD‐designed patterns. We highlight this characteristic by seeding cells on soft hydrogel microwells in the shape of the word “CELL” (**Figure** **6** and Figure S22 and Movie S3, Supporting Information). It is evident from the depth‐coded 3D image stacks that the cells can self‐organize even along these nonperiodic and asymmetric micropatterns with good fidelity.

In conclusion, our findings provide a new approach to guide the patterning and self‐organization of endothelial cells. We have defined the type and magnitude of discrete physical environmental cues that are required to drive self‐organization and substantially advanced the understanding of endothelial cooperativity during vasculogenesis.^[^ ^69^ ^]^ These findings may now be used to investigate fundamental questions in developmental biology, morphogenesis, and the etiology of diseases like cardiovascular conditions or cancer progression. In the future, geometric patterns on soft hydrogels or other biocompatible materials could also be utilized to self‐organize blood vessel networks in the desired configurations without any superimposed surface chemical modifications. This approach may provide successful solutions to the hotly pursued challenge of vascularizing tissue constructs, implants, and devices with complex and multiscale topographies.^[^ ^70^ ^]^ Since our strategy allows highly parallel, reproducible, and cost‐effective hydrogel patterning, we anticipate widespread applications in cell and tissue engineering, cell signaling, and drug discovery. Finally, our discovery that striking differences in multicellular self‐organization are caused by the combined effects of stiffness and microwell aspect ratio is of fundamental importance to understand the role of stiffness in previously observed topography‐ and curvature‐guided cell behaviors.^[^ ^36^, ^37^, ^38^, ^71^, ^72^ ^]^

## 4 Experimental Section

### Fabrication and Characterization of Micropatterned Hydrogel Substrates

The micropatterned hydrogels were fabricated as reported previously.^[^ ^39^ ^]^ Briefly, appropriate positive tone photoresists (AZ9260 and AZ5214E, MicroChemicals GmbH and SPR220, Kayaku Advanced Materials) were used depending on the microwell depth (Table S1, Supporting Information) and were exposed to UV light through a photomask to create micropatterns on a silicon wafer. After exposure and development as per the manufacturer's specifications, the micropatterns were heated beyond the photoresist glass transition temperature to induce photoresist reflow and obtain micropatterns with a curved profile. Two steps of PDMS (Sylgard 184, Dow) molding against the photoresist patterns were used to yield a final reusable micropatterned mold for gelatin. The micropatterned molds were sterilized under UV for 1 h and then treated with 1% bovine serum albumin (BSA) (Sigma‐Aldrich) for 1 h at room temperature to prevent the hydrogel from sticking to the PDMS mold. Type A gelatin from porcine skin (Sigma‐Aldrich) was used to make the hydrogels. Bloom 90–110 (low molecular weight, 20–25 kDa), Bloom 175 (medium molecular weight, 40–50 kDa), and Bloom 300 (high molecular weight, 50–100 kDa) gelatin were used resulting in soft 2 kPa, medium 8 kPa, and stiff 35 kPa hydrogel, respectively, on enzymatic crosslinking. 12.5% by weight gelatin powder was dissolved, as received, in phosphate‐buffered saline (PBS) and was sterile filtered through a 0.2 µm polystyrene membrane filter. To render the gelatin thermostable, the gelatin solution was mixed with 1 mL of sterile filtered 10 U (g gelatin)^−1^ microbial transglutaminase (mTG, prepared in PBS) (Ajinomoto). The gelatin was allowed to crosslink till it reached the desired stiffness at 37 °C after which the micropatterned area was sectioned into small disks 8 mm in diameter and heated in PBS to 60 °C for 30 min to deactivate the mTG. After the mTG was deactivated, the gelatin hydrogels were stored in PBS at 37 °C until subsequent use in the experiments.

Polyacrylamide hydrogels were prepared as per the protocol described previously.^[^ ^73^ ^]^ Briefly, 4% and 10%, wt/vol of acrylamide were mixed with 0.1% and 0.3%, wt/vol of *N,N′‐*methylene‐bis‐acrylamide crosslinker (Sigma‐Aldrich) in PBS for 2 and 35 kPa polyacrylamide hydrogels, respectively. This mixture was degassed for 10 min and 10 µL of 10% wt/vol ammonium persulfate (Sigma‐Aldrich) and 1 µL of *N,N,N′,N′*‐tetramethylethylenediamine (Sigma‐Aldrich) were added for every 1 mL of acrylamide/bis‐acrylamide solution. This mixture was poured on a silicon wafer containing the micropatterns and allowed to crosslink. The micropatterned wafer was silanized with trichloro(1*H*,1*H*,2*H*,2*H*‐perfluorooctyl)silane (Sigma‐Aldrich) to prevent the hydrogel from sticking to the micropatterns. After peeling the hydrogels, those were allowed to swell in PBS for 1 day at room temperature and were sterilized under UV for 30 min. 0.1% gelatin (Sigma‐Aldrich) or 25 µg mL^−1^ fibronectin (MilliporeSigma) was then conjugated on the micropatterned polyacrylamide hydrogels using *N*‐sulfosuccinimidyl‐6‐(4’‐azido‐2’‐nitrophenylamino) hexanoate (sulfo‐SANPAH; Thermo Fisher Scientific) in 50 × 10^−3^ m HEPES buffer. 50 µL of 1 mg mL^−1^ solution of sulfo‐SANPAH was pipetted over each 8 mm diameter hydrogel disk and exposed to 365 nm UV light for 10 min. The hydrogels were then washed with HEPES buffer and the sulfo‐SANPAH step was repeated for another 10 min, after which hydrogels were washed and incubated overnight at 4 °C with the protein solution to be conjugated.

The depth of the patterned hydrogel microwells was measured using Nikon A1 confocal microscope. The microwell patterned hydrogels were stained with 1 mg mL^−1^ fluorescein isothiocyanate (FITC) solution for 1 h at room temperature, followed by washing with PBS (15 min, 3x). A z‐stack of these FITC stained microwells was acquired, and the microwell depth was measured from the cross‐section (orthogonal view) in ImageJ (NIH).

### Mechanical Characterization

The bulk mechanical properties of the hydrogels were measured using oscillatory shear rheometry and the local mechanical properties using AFM. The Anton Paar MCR 302 rheometer was used to measure the shear modulus and stress relaxation. Hydrogel disks (25 mm diameter, ≈3 mm thickness) hydrated with PBS were loaded between a piece of 180G sandpaper and a 25 mm sandblasted plate probe and all the measurements were conducted at 37 °C. The storage and loss moduli was first measured at a constant strain of 0.5% over 20 min, followed by a strain sweep at a constant frequency of 1 Hz and 0.5 N normal force. Young's modulus was estimated from the shear modulus using a Poisson's ratio of 0.5.

Next, the local Young's modulus of the micropatterned hydrogels was measured using a Bruker Catalyst AFM which sat atop a Zeiss Observer inverted microscope. An AFM probe with a tip diameter of 5.13 µm and a spring constant of 0.188 N m^−1^ (Bruker) was used to indent the hydrogel surface and determine Young's modulus. Measurements were conducted on three samples for the soft and stiffest hydrogel and two samples for the medium stiffness hydrogel to collect 25 force–displacement curves (5 × 5 array, 10 µm spacing) on at least two different regions of the hydrogel sample. The force–displacement curves were analyzed using a Hertzian model in the Nanoscope analysis software to obtain Young's moduli.

### Scanning Electron Microscopy

Samples were prepared for SEM by two methods: lyophilization (to visualize the pores) and glutaraldehyde fixation (to visualize the surface). For lyophilized hydrogels, the samples were first frozen at −80 °C for 2 days which were then freeze‐dried for 3 days. The dried samples were sliced and 5 nm of gold was sputter‐coated on the cross‐section to visualize the pores using a SEM (JEOL). To view the surface topography of the microwells, the micropatterned hydrogels were fixed with a fixative containing 3.0% formaldehyde (Electron Microscopy Sciences) and 1.5% glutaraldehyde (Electron Microscopy Sciences) in a 0.1 m NaCacodylate buffer (pH 7.4), at room temperature for 1 h. The samples were then washed and post‐fixed in 1% osmium tetraoxide (Sigma‐Aldrich) on ice for 1 h. These samples were then dehydrated using a graded series of ethanol (50%, 70%, 95%, 100%) and three 15 min washes in fresh 100% ethanol at room temperature. The ethanol was then replaced with a graded series of hexamethyldisilazane (HMDS, Electron Microscopy Sciences) (50%, 75%, and 100%) followed by two 30 min washes in fresh 100% HMDS. After the HMDS wash, the HMDS was allowed to evaporate at room temperature to completely dry the samples. These dried samples were sputter‐coated with gold and the microwells were visualized at an angle using a SEM (JEOL).

### Cell Culture, Transduction, Seeding, and Live‐Cell Imaging

HUVECs (ScienCell) were cultured in endothelial cell growth media (PromoCell) with 1% penicillin‐streptomycin. The 8 mm micropatterned hydrogel disks were placed inside glass cloning rings (Fisher Scientific), which fit snugly around the hydrogels to hold the cells and media.^[^ ^39^ ^]^ The HUVECs were seeded at the desired cell density on the hydrogels and their growth was monitored for 24 h. The cells were treated with the relevant pharmacological agent and its corresponding vehicle controls for experiments involving alteration of cytoskeletal stiffness. Y‐27632 (Cell Signaling Technology) was included with the cells upon seeding, whereas Calyculin A (Sigma‐Aldrich) was added 4 h following cell seeding.

HUVECs were transduced with rAV‐CMV‐LifeAct Adenoviral Vectors (Ibidi) to express LifeAct GFP. The viral vector was mixed with media and incubated with the cells for 8 h 1 day before seeding the cells in the microwells. After 8 h, the virus‐containing media was replaced with fresh media and the cells were checked for LifeAct GFP expression the next day.

The cell migration was recorded in the microwells at regular intervals for more than 18 h using a × 10/0.45 NA Ph1 objective on an inverted Nikon Eclipse Ti microscope (Nikon) or an A1 confocal microscope (Nikon). Both microscopes had automated controls (NIS‐Elements, Nikon) and were equipped with an incubation chamber. The cell migration tracks were generated using the MTrackJ plugin in ImageJ (NIH) and cell proliferation was determined by manually counting cell divisions occurring in the microwells.

### Immunofluorescence Staining

The cells seeded in hydrogel microwells were fixed with 3% paraformaldehyde (Sigma‐Aldrich) for 20 min, permeabilized with 0.1% Triton X‐100 (Sigma‐Aldrich) for 15 min, and blocked with 2% BSA (Sigma‐Aldrich) or 5% Normal Horse Serum (Invitrogen) in PBS for 1 h to prevent nonspecific binding of the antibody used. After blocking, the samples were incubated for 1 h at 37 °C with an appropriate primary antibody and then incubated at room temperature with a secondary antibody and fluorescent probe for 1 h. All antibodies and fluorescent probes were diluted in 0.1% BSA in PBS. Primary antibodies included rabbit anti‐VE‐cadherin antibody (160840, Cayman Chemicals, 1:150) and rabbit anti‐Phospho‐Paxillin (69363, Cell Signaling, 1:100). The secondary antibody was goat anti‐rabbit antibody conjugated with Alexa Fluor 488 (A‐11034, Invitrogen, 1:150 for VE‐Cadherin and 1:100 for Paxillin). Actin was labeled with Alexa Fluor 488 Phalloidin (A12379, Invitrogen), or Rhodamine Phalloidin (R415, Invitrogen). Nuclei were labeled with 4',6‐diamidino‐2‐phenylindole, dihydrochloride (DAPI) (D1306, Invitrogen). The cells were labeled directly on the micropatterned hydrogels in the glass cloning rings to avoid disrupting the cell organization and were stored in PBS.

### Image Acquisition and Analysis

The glass rings were detached enclosing the hydrogel sample for imaging the cells in the microwells and the hydrogel samples were either inverted on glass‐bottom dishes (MatTek Corporation) with the patterned surface close to the objective lens or were mounted in FluorSave (Millipore). A Nikon A1 confocal microscope was used to acquire the z‐stacks and the stacks were analyzed using ImageJ (NIH) and Imaris (Bitplane) software.

The confocal z‐stacks were used to quantify the *DR* by measuring the distance between the microwell center and the center of the nucleus. A 2D maximum intensity projection of the 3D stack was generated in ImageJ and the distance between the center of the nuclei and the center of the projected microwell was measured in ImageJ. Cell area was measured manually by outlining the cell body, identified with VE‐cadherin staining, in the maximum intensity projection generated above. Since the cell size was measured using a projected image, in some instances, cells appeared to be overlapping each other (cells moving on top of each other in the different *z*‐planes, but not in contact). In such cases, the 3D stack was split in the *z*‐direction before generating a projection to avoid overlap.

For actin intensity measurements, an average intensity projection of the 3D stack was first generated. The actin intensity across the diameter of the projected microwell was then measured at 30° intervals for each microwell. The intensity values for each point along the diameter (*x*) were then averaged over multiple microwells and plotted as a line graph. To generate the actin intensity heat map, a region of interest (ROI) corresponding to the microwell and the flat region between the microwells was first defined. This ROI was then used to extract individual microwells from the average intensity projection created above. The extracted images were then aligned, stacked, and an average intensity projection was generated to yeild the distribution of actin in the microwell. This projection was used to create the actin intensity heat map.

To measure paxillin‐containing FAs, the confocal *z*‐stack was used to convert the paxillin signal into 3D surfaces using the *Surfaces* feature in Imaris and the noise was eliminated by limiting the minimum surface area to 0.5 µm^2^. Next, the paxillin surfaces rendered by the software for each cell were examined and surfaces that were not at the cell–substrate interface were manually deleted. FA surfaces were then categorized into two groups according to their location in the microwell: microwell center or microwell edge. The surface area of the paxillin‐containing FAs was then extracted for further analysis and presentation.

For VE‐cadherin, the 2D maximum intensity projection of the VE‐cadherin‐signal‐containing 3D stack was used to identify the junction region. An average intensity projection of the same 3D stack was then generated and the junction area identified from the maximum intensity projection was used to quantify the fluorescence intensity of VE‐cadherin junctions over the image field using ImageJ. These measurements were verified in 3D using Imaris in a process similar to that described for measuring the paxillin‐containing FAs.

### Brillouin Microscopy Measurements and Analysis

Brillouin microscopy is a noncontact spectroscopic method to probe the mechanical properties of materials with high spatial resolution in 3D.^[^ ^46^, ^47^ ^]^ Recently, it has been applied to map mechanical properties of biological materials at high resolution, including hydrogels, cells, and entire organisms.^[^ ^48^, ^49^ ^]^ The Brillouin shift of the hydrogels was measured using a two‐stage Virtually Imaged Phase Array (VIPA) spectrometer as described previously.^[^ ^47^, ^74^ ^]^ In brief, the sample was illuminated with 660 nm laser light through a 60x, 0.7NA objective (Olympus). Backscattered light (at 180°) was collected through the same objective and coupled through an optical fiber into a two‐stage VIPA spectrometer. This configuration ensured that the Brillouin microscope operated in a confocal regime. The optical spectra collected in each confocal volume were analyzed by the two‐stage VIPA spectrometer which were recorded using an EMCCD camera (Andor).

The samples, immersed in PBS in a glass‐bottom imaging dish (Ibidi), were placed on the motorized stage of the Olympus IX81 microscope and vertical confocal slices (*z*‐direction) were scanned at multiple points across the microwell (Figure 2C). One spectrum (50 ms exposure time) was recorded for each pixel (0.5 µm × 0.5 µm) in the acquired images. The resolution of the Brillouin microscope was 0.6 µm in the *x*–*y* direction and 1.9 µm in the *z*‐direction. The Brillouin shift of the light in the confocal volume was independent of the slope of the microwell surface. It was confirmed that the transition in the *z*‐direction of the hydrogel's measured shift to the PBS buffer's shift was consistent across multiple locations on the microwell surface and that it was corresponded to the *z*‐resolution of the microscope (2 µm) (Figure S9B, Supporting Information). Data collection was performed through a custom LabView program. Each spectrum was fitted with a Lorentzian function using the least‐squares method to localize Lorentzian‐shaped Brillouin scattering peaks (MATLAB). The spectral dispersion (GHz/pixel) and free spectral range (FSR) of the Brillouin spectrometer were calibrated by measuring two liquids of known Brillouin shift (water and methanol) prior to measuring the samples. The Brillouin shift's spectral precision was typically 8 MHz (estimated as the standard deviation of the Brillouin shift of PBS shift in each image).

To testthe homogeneity of the microwell surface, the frequency shifts were extracted every 5 µm along the microwell diameter (*x*) and up to 2.5 µm inside the hydrogel from the surface (*z*). The shift was then averaged in the *z*‐direction at each *x* in the microwell to obtain the plot in Figure 2D.

### Statistical Analysis

The experimental data were analyzed for statistical significance wherever required and the data were plotted using OriginPro 2021(OriginLab) software. The surface area distribution of paxillin‐containing FAs was tested for normality using the Kolmogorov–Smirnov test and was found to be right‐skewed. The Kruskal–Wallis one‐way analysis of variance (ANOVA) was used to compare the different groups (2 kPa edge, 2 kPa center, 35 kPa edge, and 35 kPa center). It was concluded from the Kruskal–Wallis one‐way ANOVA that the groups were significantly different. The median FA size was then selected for each cell in respective conditions and statistically compared using one‐way ANOVA followed by Tukey's mean comparison. The VE‐cadherin data were compared using the unpaired Student's *t*‐test (two‐tailed), and the cell area data were compared using the Mann–Whitney test.

## Conflict of Interest

The authors declare no conflict of interest.

## Author Contributions

G.J.P., L.H.R., and D.H.G. conceived the study and designed the experiments. G.J.P. performed the experiments and data analysis under the supervision of L.H.R. and D.H.G. S.G. helped with schematics and data analysis. J.T. and S.X.S. formulated the mathematical model. M.N. and G.S. performed the Brillouin imaging. G.J.P., L.H.R., and D.H.G. wrote the manuscript with input from all the authors.

## Supporting information

Supporting Information

Supplemental Movie 1

Supplemental Movie 2

Supplemental Movie 3

## Data Availability

The data that support the findings of this study are available in the supplementary material of this article.
