# Supplementary material for: Directing Multicellular Organization by Varying the Aspect Ratio of Soft Hydrogel Microwells
Source: Adv Sci (Weinh). 2022 Apr 17;9(17):2104649. doi: 10.1002/advs.202104649 (PMC9189654; doi:10.1002/advs.202104649)
Supplement: Supplementary file 1 — Supporting Information [file ADVS-9-2104649-s001.pdf]

ADVANCED SCIENCE
Open Access

Supporting Information

for *Adv. Sci.*, DOI 10.1002/advs.202104649

Directing Multicellular Organization by Varying the Aspect Ratio of Soft Hydrogel Microwells

Gayatri J. Pahapale, Jiaxiang Tao, Milos Nikolic, Sammy Gao, Giuliano Scarcelli, Sean X. Sun, Lewis H. Romer and David H. Gracias**

Supporting Information

Directing multicellular organization by varying the aspect ratio of soft hydrogel microwells

Gayatri J. Pahapale, Jiaxiang Tao, Milos Nikolic, Sammy Gao, Giuliano Scarcelli, Sean X. Sun, Lewis H. Romer, David H. Gracias**

*Corresponding author. Email: lromer@jhmi.edu (L.H.R.); dgracias@jhu.edu (D.H.G.)

Supporting Text: Notes S1 and S2

Figures S1 to S22

Tables S1 to S3

Movies S1 to S3

Supporting Text

Note S1: General mathematical model description

To study the mechanism of multicellular organization in microwells, we developed a mathematical model based on force interactions between the cells and substrate. As illustrated in Figure 5A and Figure S21A, the model consists of cells in a microwell projected on a 2D x-z plane. The cell dynamics predicted by the 2D model can be restored to 3D geometries by considering the 2D geometry as a crosssection of a 3D shape along appropriate cell orientation. For hemispherical microwells, the translation is straightforward due to symmetrical geometric properties and only requires adjusting the steady-state cell length. In a cylindrical microwell, the 3D model would have to consider aspect ratios along two planes. Therefore, the force-balance mechanism explained by the 2D model provides insights into the observed cellular organization, regardless of the 3D microwell shape (i.e., cylinder vs. hemisphere). We assume the cells to be moving upwards where the cell is modeled as a single-line element with all the forces only acting on each cell's endpoints.

The geometric shape of the microwell, along the cell movement dimension, with crossectional depth H and radius R can be described as:

$$z = H - H\sqrt{1 - \frac{r^2}{R^2}} \tag{1}$$

where z is the local height which ranges from 0 to H and r is the local radius at z, ranging from 0 to R. $(r(z = 0) = 0; r(z = H) = R)$. The region outside the microwell is infinitely flat, or: $z = H$ for $r > R$. The angle between the local tangential line (red dotted line in Figure S21A) and x-axis can be described as: $\tan\theta = \frac{dz}{dr} = \frac{Hr}{R^2\sqrt{1-\frac{r^2}{R^2}}}$ for $r < R$; and $\tan\theta = 0$ for $r \geq R$, or $z = H$. This simplified 2D geometry can illustrate a more complex 3D microwell curvature along the cell's movement direction, as the local curvature is a function of the first and second derivative of r in respect to z.

By considering cytoskeletal tension (F_k), which regulates the cell shape and size, cell-substrate interaction, which is the frictional force (F_η), and cell-cell interaction (F_w), we can write the force-balance equation at i^{th} node of jth cell, $\mathbf{x}_{j,i}$, as:

$$\eta\dot{\mathbf{x}}_{j,i} = -\frac{d}{d\mathbf{x}_{j,i}}\left[\frac{k}{2d_0}\left(d_j - d_0\right)^2\right] + A\mathbf{f}_{j,i} + F_0\sum_{m,k}\mathbf{w}\left(\mathbf{x}_{j,i}, \mathbf{x}_{m,k}\right) \tag{2}$$

where each cell is described by two nodes (i = 1,2). We describe the intracellular cytoskeletal tension using spring potential with the stiffness of k. The equilibrium length of the cell is d_0, and the current length of the cell j is $d_j = |\mathbf{x}_{j,1} - \mathbf{x}_{j,2}|$ The cell constantly generates active protrusion and contraction forces, $\boldsymbol{f}_{j,i}$. This force, scaled by a constant A, follows the random Gaussian distribution with zero mean and is inversely proportional to the cell length, d_j: $\mathbf{f}_{j,i} = \frac{E(t)}{d_j}$, $< E(t_1)E(t_2) > = \sigma^2\delta(t_1 - t_2)$ where σ is the standard deviation of the Gaussian distribution. This force is along the local tangential direction of the substrate. Interaction between the ith node of the jth cell and the kth node of the mth cell is $\boldsymbol{w}(\mathbf{x}_{j,i}, \mathbf{x}_{m,k})$, and follows the van der Waals relation. This interaction becomes repulsive when the distance between the two nodes, $|\mathbf{x}_{j,i} - \mathbf{x}_{m,k}|$, is less than s_0 (see Table S3) and attractive if the distance is greater than s_0 and fades to zero when $|\mathbf{x}_{j,i} - \mathbf{x}_{m,k}| \rightarrow \infty$. To circumvent the singularity caused by the nature of van der Waals relation when $|\mathbf{x}_{j,i} - \mathbf{x}_{m,k}| \rightarrow 0$, we fitted a continuous stepwise function to the van der Waals equation so that $\boldsymbol{w}(\mathbf{x}_{j,i}, \mathbf{x}_{m,k})$ goes from -1 to 1 (Figure S21B). $\boldsymbol{w}(\mathbf{x}_{j,i}, \mathbf{x}_{m,k})$ is scaled by a constant F_0, and is along the linear direction between these two nodes (green dotted line in Figure S21A). F_0 represents cell-cell interaction strength, a function of VE-cadherin, or other intracellular protein activities. Therefore, our model covers cell-cell attractions at long distances and cell-cell repulsion at short distances. We assume the cell-cell interaction is only significant between the top and bottom nodes of the cell since the distances between any other two nodes are much further than s_0. η is the frictional coefficient between the cell and substrate. The right-hand side of the force balance (Equation 2) describes the residual force resulting from tension, random protrusions and contractions, and cell-cell interaction, and the left-hand side describes the sliding frictional force between the cell and the substrate. This force balance equation indicates the unbalanced force at each node compensated by the local sliding motion along the microwell.

Numerical Results

Since the cells can only move along the tangential direction, we can simplify the system by considering only the tangential direction of Equation 2 for each cell node. The dynamic equations for cells 1 (top) to N (bottom) can then be written as:

$$\dot{x}_{j,1} = -\frac{k}{d_0\eta}(d_j - d_0)\cos\left(\phi_j - \theta(x_{j,1})\right) + \frac{A}{d_j\eta}E_{j,1}(t) - \frac{F_0}{\eta}w_{j-1,2\rightarrow j,1}\cos\left(\beta_{j-1,j} - \theta(x_{j,1})\right)\left(1 - \delta(j,1)\right);$$

$$\dot{x}_{j,2} = \frac{k}{d_0\eta}(d_1 - d_0)\cos\left(\phi_j - \theta(x_{j,2})\right) + \frac{A}{d_j\eta}E_{j,2}(t) + \frac{F_0}{\eta}w_{j,2\rightarrow j+1,1}\cos\left(\beta_{j,j+1} - \theta(x_{j,2})\right)\left(1 - \delta(j,N)\right) \quad (3)$$

as j ranging from 1 to N. φ_j denotes the angle between j^{th} cell ($\mathbf{x}_{j,2}$ - $\mathbf{x}_{j,1}$) and the x-axis. φ_j – $\theta(\mathbf{x}_{j,i})$ denotes the orientation of the cells relative to the microwell's tangential direction at the ith node of the jth cell: $\mathrm{x}_{j,i}$. $\beta_{j-1,j}$ is the angle between the line connecting the two cell nodes ($\mathbf{x}_{j,1}$ - $\mathbf{x}_{j-1,2}$) and the x-axis. We used a MATLAB package to generate random $E_{1,1}$, $E_{1,2}$, $E_{2,1}$, $E_{2,2}$, $E_{3,1}$, $E_{3,2}$,....., $E_{N,1}$ and $E_{N,2}$ independently from the normal distribution with zero mean and pre-defined standard deviation σ at each step. $\delta_{a,b}$ is a delta function ($\delta_{a,b} = 1$ if a=b), indicating no intracellular interaction at the top. To simplify the model, we solve Equation 3 for a 2-cell system.

We computed Equation 3 for 2,000 timesteps with a time increment of dt = 0.01 and ran the entire simulation over 200 times to statistically quantify the steady-state multicellular organization in the microwell. We determined whether the cells were located edge or to the center by defining a height ratio: $\frac{z_0}{H}$, in which z_0 is the average z coordinate of the cell's center point after each of the 1,000-time iteration for each simulation. If $\frac{z_0}{H}$ is close to 1, the cells are more likely to be at the microwell edge, and if $\frac{z_0}{H}$ is closer to zero, the cells are more likely to be towards the center. We define cells to be organized on edge if $\frac{z_0}{H} \geq 0.8$, and to the center if $\frac{z_0}{H} \leq 0.2$. We then quantified the ratio between the probability of cells staying on the microwell's edge and the probability of the cells staying towards the center, N_{edge} / N_{center} based on all 200 simulations. We define N_{edge} / N_{center} as DR in the main text.

The cells are more likely to stay at the microwell center in the shallower microwells but relocate to the edges of deeper microwells. With the correct set of parameters, our simulation results can predict cellular organization as observed experimentally (Figure 5C). We could also predict the sensitivity of multicellular organization to geometry in response to cytoskeletal contractility (or tension) influenced by substrate stiffness and cell-cell interactions using this force balance model (Figure 5C and D), and these predictions are consistent with experimental findings (Figure S14, S15, and S17). Solving Equation 3 for a 3 or 4-cell system yielded similar trends as the 2-cell system (Figure S21E-H). The geometric setting of the experiment ($d_0 \sim \frac{R}{2}$) allows a maximum of 3 or 4 cells in one microwell (Figure S21I-J). However, the real experimental setting is equivalent to applying periodic boundary conditions to Equation 3, as cells that are pushed out would enter a neighboring microwell. Therefore, we can extend our findings to a monolayer seeded on a periodically microwell-patterned substrate.

The observed spatial localization can be explained by the force-balance feature of the system, caused due to the geometry of the microwell. Cells rely on cytoskeletal activities and forces to move and balance extracellular forces, including cell-cell interactions and cell-substrate friction. Since the cytoskeletal tension is not precisely along the microwell's local tangential direction, the magnitude of tangential components of cytoskeletal tension depends on the cell's orientation relative to the microwell's tangential direction. The unbalanced part of the force should be balanced by friction, and drives the cell movement along the microwell. Therefore, the cell is less likely to move when the cytoskeletal tension is more likely to balance the extracellular forces. This balance is most likely to occurs at microwell positions where $|\varphi_j - \theta_{j,i}| \sim 0$. According to the geometric calculation, $|\varphi_j - \theta_{j,i}|$ is close to zero around the center of shallower microwells or along the edge of deeper microwells (Figure S21C). Thus, when the cell-cell interactions are increased, higher tangential intracellular cell tension components are required to balance the increased interaction, achievable on the microwell edge. Similarly, when the cell contractility (k) is decreased due to a decrease in substrate stiffness, the cell again moves to the edge where a higher tangential component of tension is achievable to balance the other forces of the system. Further, to quantify the unbalanced force which drives the cell movement up and down, we calculated the mean vertical velocity of the cells when they are located at the center and top edge of the microwell, respectively, and averaged them over all the nodes, v_z. The vertical velocity indicates the vertical component of the frictional force, which drives the vertical movement of the cells. As shown in (Figure S21D), when cells are seeded in deeper microwells, higher vertical velocity towards the center and lower vertical velocity towards the top edge is predicted compared to cells seeded in the shallower microwell, indicating a higher probability of cells moving towards the top edge in deeper microwells ($\varepsilon < 25$), and towards the center in shallower microwell ($\varepsilon > 25$).

Note S2: Note on choice of enzymatic crosslinking vs photocrosslinking.

In general, there are two popular ways to crosslink gelatin: Photo or chemical crosslinking. Our laboratory has extensive expertise and published papers using photocrosslinked GelMA.[1,2] While our previous work included fabricating flat sheets crosslinked in a chamber constructed using glass, the hydrogels prepared in the current work are micropatterned and rely on crosslinking the hydrogel while molding against a PDMS mold. We observed that the GelMA did not crosslink uniformly when exposed to UV light on the micropatterned PDMS mold with either the Igracure 2959 UV light photoinitiator or a Ruthenium visible light photoinitiator. Due to incomplete crosslinking, the gel surfaces tore and were damaged during peeling after photocrosslinking. This insufficient crosslinking on PDMS and other oxygen-permeable

substrates, when photocrosslinked in air, is a well-known problem and has been observed with many hydrogels that are crosslinked via free-radical polymerization in the case of Irgacure 2959.[3] In free-radical polymerization, the initiator molecules form radicals, initiating the reaction with monomers, which then propagates, to form polymer chains. In the presence of oxygen, the active radicals are scavenged, leading to dead chain ends and inhibiting radical polymerization yielding a partially crosslinked hydrogel.

Microbial Transglutaminase (mTG) is a well-known chemical crosslinker used to crosslink gelatin for various applications.[4-8] mTG-based crosslinking is not significantly affected by the presence of oxygen, and the crosslinked hydrogels have uniform mechanical properties on the surface and the bulk as long as mTG is mixed correctly with the pregel solution. The reaction does not lead to any harmful byproducts, and the byproducts produced are removed from the gel when the enzyme is deactivated and washed at 60°C. Also, transglutaminase crosslinks the glutamine and lysine amino acids of gelatin, thus keeping the cell adhesive RGD motif intact.[9]

Additional References

1. H.R. Kwag,* J. V. Serbo,* P. Korangath, S. Sukumar, L.H. Romer, D.H. Gracias, *Tissue Eng. Part C* **2016**, 22, 398.
2. R. Shi, J. Fern, W. Xu, S. Jia, Q. Huang, G. Pahapale, R. Schulman, D.H. Gracias, *Small* **2020**,16, 2002946.
3. R. Simic, J. Mandal, K. Zhang, N. D. Spencer, *Soft Matt*er **2021**.
4. A. Paguirigan, and D. J. Beebe. *Lab Chip,* **2006,** 6.3, 407.
5. C. W. Yung, L. Q. Wu, J. A. Tullman, G. F. Payne, W. E. Bentley, T. A. Barbari, *J Biomed Mater Res A*, **2007** 83(4), 1039.
6. Z. Yang, and J. Tramper. *Trends Biotechnol.* ***2008,*** 26.10, 559.
7. S. Fuchs, M. Kutscher, T. Hertel, G. Winter, M. Pietzsch, C. Coester, *J. Microencapsul.* **2010**, 27(8), 747.
8. M. Zhou, B. H. Lee, Y. J. Tan, L.P. Tan, *Biofabrication*, **2019**, 11(2), 025011.
9. Y. Zhu, A. Rinzema, J. Tramper, J. Bol, *Appl. Microbiol. Biotechnol*. **1995**, 44(3), 27.

Supporting Figures

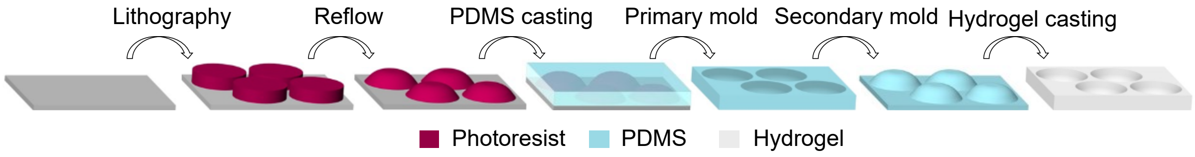

Figure S1: Process flow used for creating hydrogel microwells using reflow lithography and replica molding.

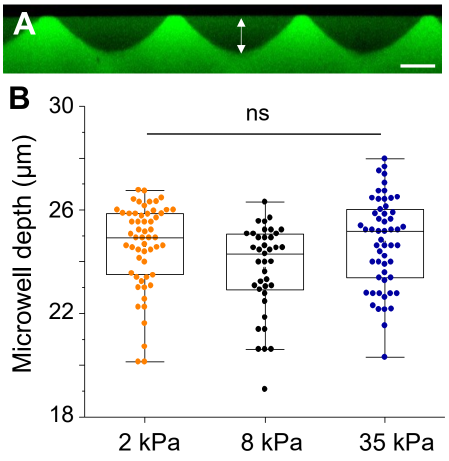

Figure S2: Microwell depth is similar in substrates of different stiffness. (A) Representative cross-sectional view of the microwells generated using a 3D confocal stack of fluorescein-stained hydrogels. Scale bar = 25 µm. (B) Plot depicting the depth of 2 kPa, 8 kPa, and 35 kPa microwells. Data presented are quantified from at least 30 microwells from three independent experiments. ns signifies $P > 0.05$ as calculated using one-way ANOVA followed by Tukey's mean comparison.

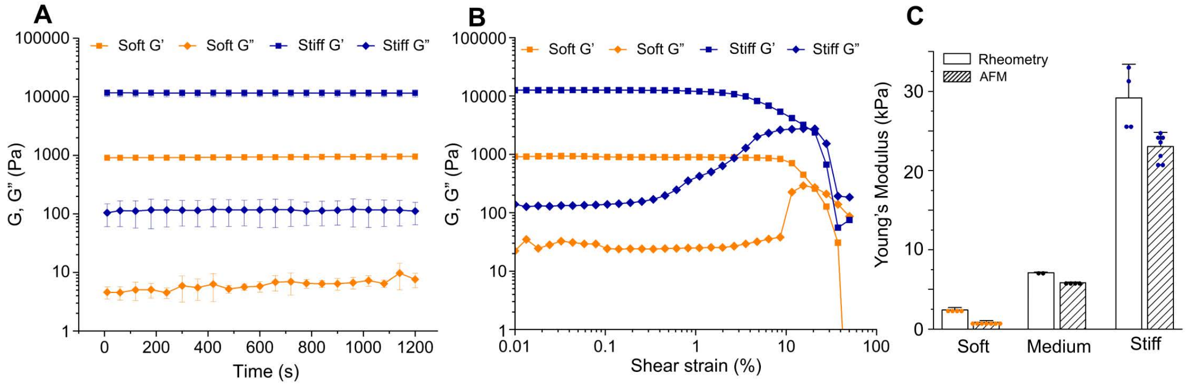

Figure S3: Mechanical characterization of hydrogels using rheometry and AFM. Shear storage (G') and loss (G") modulus during (A) time sweep under constant strain of 0.5% and frequency 1 Hz and (B) amplitude sweep under constant frequency 1 Hz, indicating that the hydrogel properties do not change over time and under strain. (C) Young's moduli for the hydrogel was estimated from the shear moduli (rheology) and AFM. These measurements signify that the trend observed in bulk and local mechanical properties is the same.

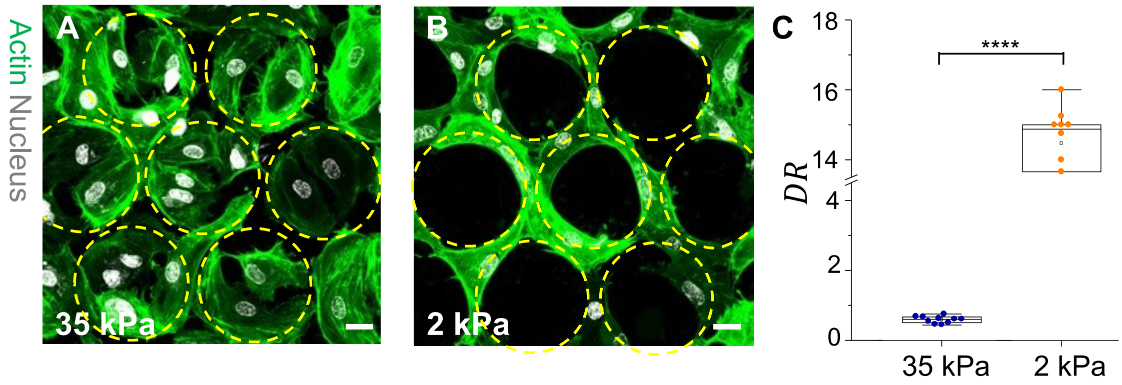

Figure S4: Multicellular self-organization in polyacrylamide hydrogel microwells is similar to that on gelatin microwells. (A, B) Confocal images showing the top view of HUVEC labeled for actin (green) and nucleus (gray) on stiff 35 kPa and soft 2 kPa polyacrylamide hydrogels 24 h after seeding. Scale bar = 25 µm. (C) Plot depicting the distribution ratio (*DR*) on 35 kPa and 2 kPa polyacrylamide microwells. Data presented were collected from 10 sample regions from three independent experiments. ****P <0.0001 Student's t-test (two-tailed).

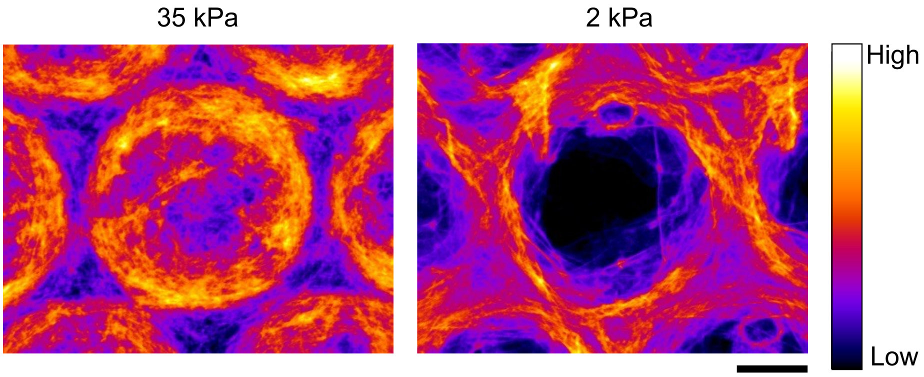

Figure S5: Actin fluorescent intensity heat maps for soft 2 kPa and stiff 35 kPa microwells indicating the approximate location of cells in microwells. Each heat map was generated from at least 30 microwells. Scale bar = 25 µm.

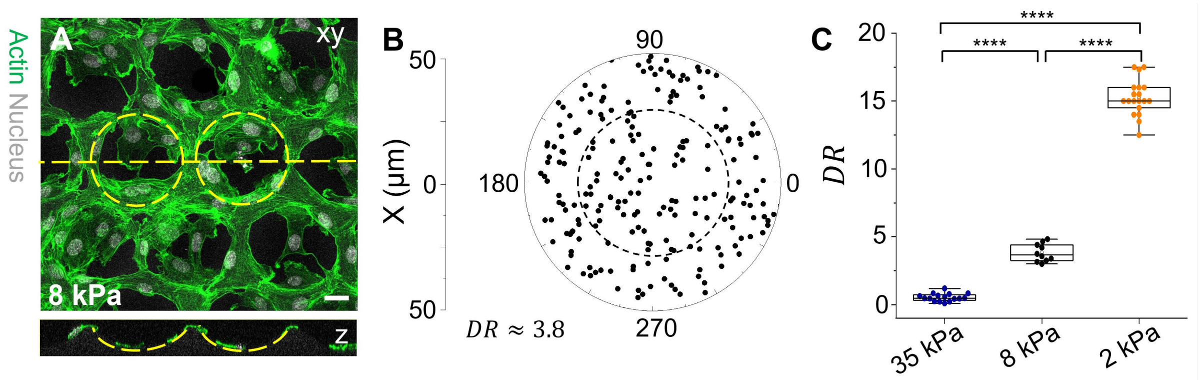

Figure S6: (A) Confocal images showing the top (xy) and side-views (z) of actin (green) and nucleus (gray) stained cells 24 h after seeding in 8 kPa. Scale bar = 25 µm. (B) Polar plot depicting the distribution of cells in the microwells quantified by measuring the distance of the nucleus from the center of the microwell. This plot was generated from measurements using >150 cells from three independent experiments. (C) Plot depicting that the distribution ratio (*DR*) on 35, 8, and 2 kPa microwells are significantly different. Data presented were collected from >12 sample regions from three independent experiments. ****P <0.0001 calculated by one-way ANOVA followed by Tukey's mean comparison.

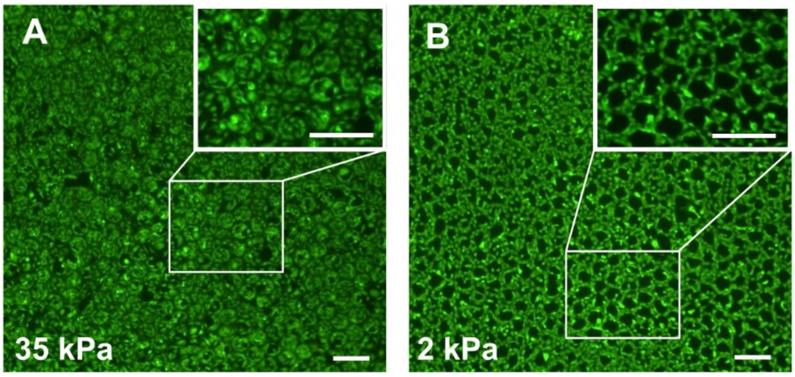

Figure S7: Cell self-organization over large areas indicates that the phenomenon and microwell hydrogels are spatially uniform. (A, B) Large area (1mmx1mm) fluorescence microscopy images of cells stained with Calcein AM in stiff 35 kPa (A), and soft 2 kPa (B) gelatin microwells 24 h after seeding. Scale bar = 200 μm.

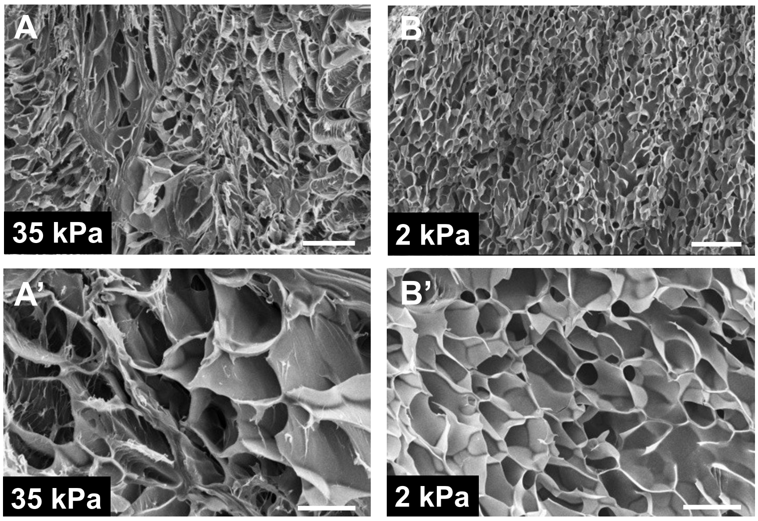

Figure S8: SEM micrographs of lyophilized gelatin hydrogel crosssections for (A, A') stiff 35 kPa with a pore size of 60 ± 28 μm and (B, B') soft 2 kPa hydrogels with a pore size of 45 ± 20 μm at two length scales. Scale bar = 200 μm for A, B and Scale bar = 50 μm for A', B'.

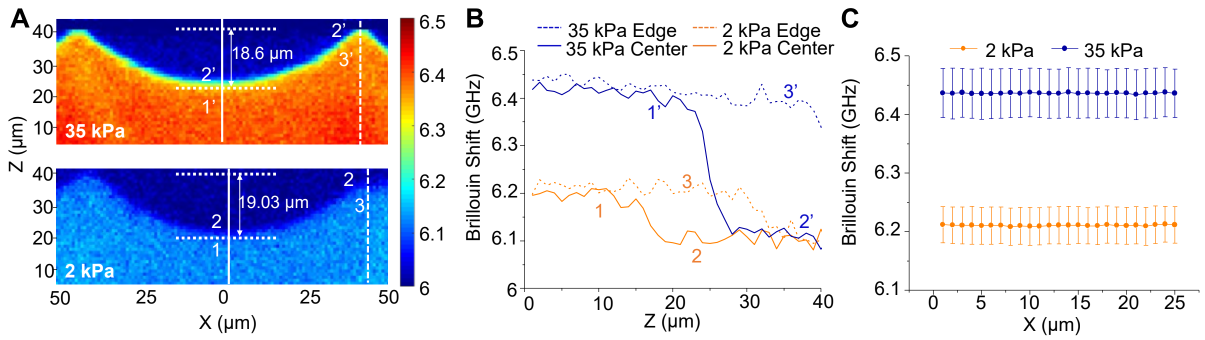

Figure S9: Brillouin imaging indicates homogenous mechanical properties in 35 kPa and 2 kPa hydrogels. (A) Representative Brillouin images showing the microwell crosssection for stiff 35 kPa and soft 2 kPa microwells. Dotted horizontal lines indicate the depth of the microwells. Heat map scale indicates Brillouin Shift in GHz. (B) Representative plot depicting the Brillouin frequency shift along the z-direction at two positions; microwell center as indicated by the solid line and the microwell edge indicated by the dashed line. Points 1',3' (35 kPa) and 1,3 (2 kPa) are inside the hydrogel, whereas points 2' (35 kPa) and 2 (2 kPa) are in PBS. The transition from the higher constant value of Brillouin shift to ~ 6.1 GHz (Brillouin shift of the surrounding PBS) corresponds to 2 µm, which is the axial resolution of the objective we used to measure Brillouin shift. (C) Brillouin shift on the surface of flat hydrogels. The Brillouin shift on flat hydrogels is similar to that in the microwells and the flat region between the microwells. Data presented are mean±SEM and were collected for n = 3 samples in each condition.

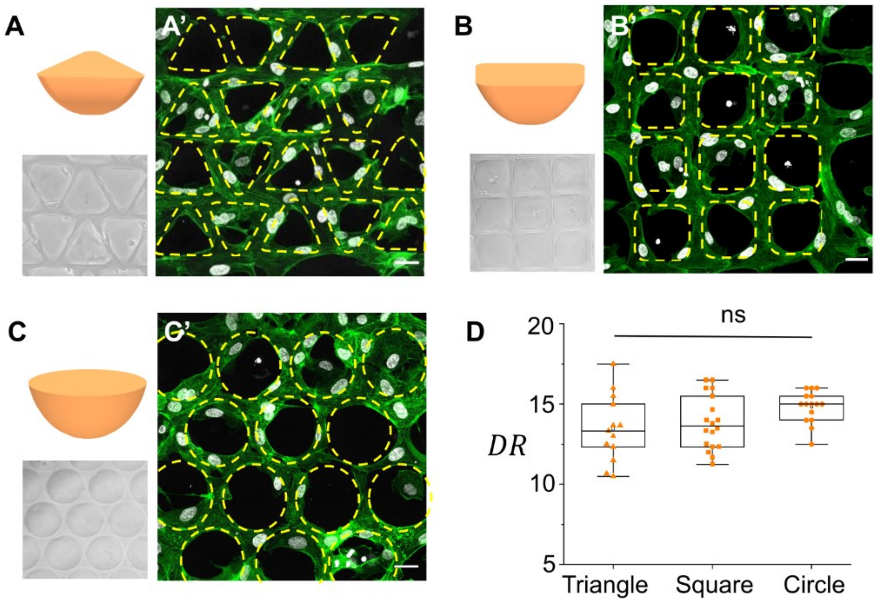

Figure S10: Cell organization on soft hydrogels is independent of the microwell shape. (A, B, C) Schematic and optical images of microwells with triangular, square, and circular perimeter. (A', B', C') Confocal images showing the top view of cells in microwells corresponding to (A, B, C). Scale bar = 25 µm. (D) Box-whisker plot depicting the distribution of cells (*DR*) in microwells of different shapes. Data presented are quantified for > 10 samples from three independent experiments. ns signifies $P > 0.05$ as calculated using ANOVA followed by Tukey's mean comparisons.

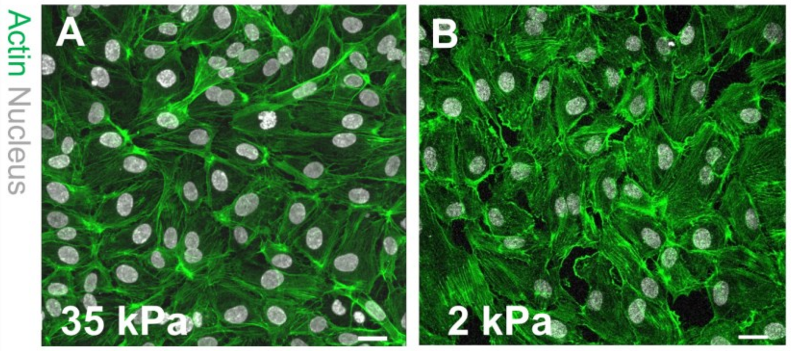

Figure S11: Confocal images showing the top (xy) view of actin (green) and nucleus (gray) stained cells 24 h after seeding on flat hydrogels. Scale bar = 25 µm.

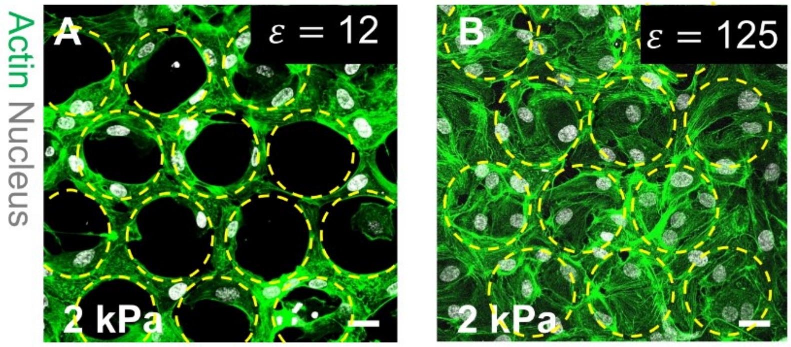

Figure S12: Multicellular organization in 2 kPa microwells with different microwell aspect ratios is strikingly different. (A, B) Confocal images showing the top view of cells stained for actin (green) and nucleus (gray) in soft 2 kPa microwells with depth 20 µm, ε = 12 (A), and 2 µm, ε = 125 (B) 24 h after seeding. Scale bar = 25 µm. Yellow dashed circles depict the microwell location.

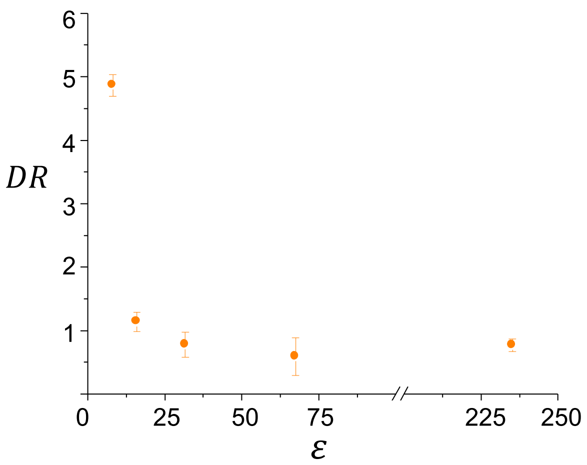

Figure S13: Multicellular organization in larger microwells follows a trend similar to 250 µm perimeter microwells. Plot depicting extent of self-organization in soft 2 kPa microwells with a perimeter of 470 µm as the aspect ratio (ε) increases (or depth decreases). The data presented are mean±SD quantified from 100 cells for each aspect ratio and repeated three times.

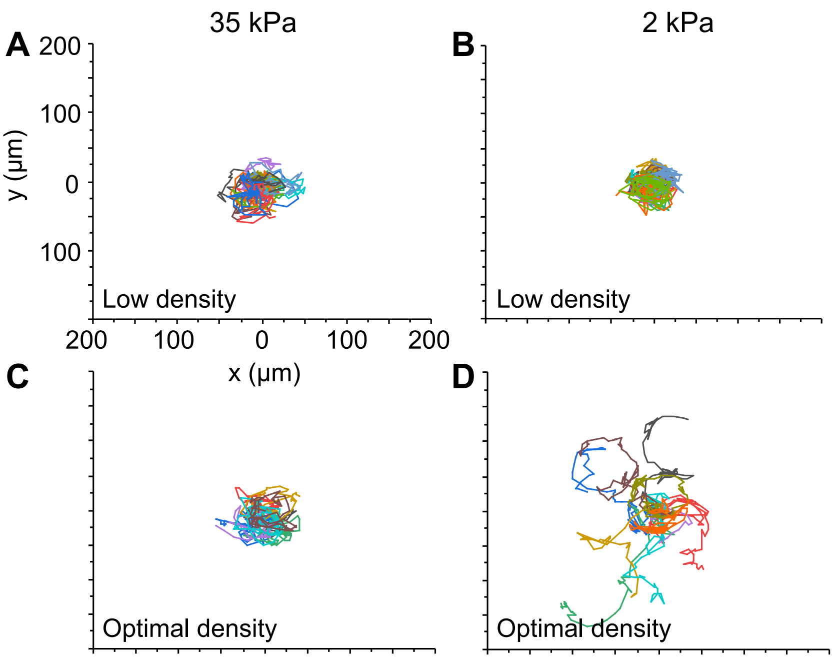

Figure S14: Migration of cells with and without neighbors in the microwell. (A-D) Cell migration tracks in stiff 35 kPa and soft 2 kPa microwells at low cell density (A, B) and optimal cell density (C, D). At optimal seeding density, each microwell has 3-5 cells allowing them to make cell-cell adhesions, whereas at low density each microwell has one cell.

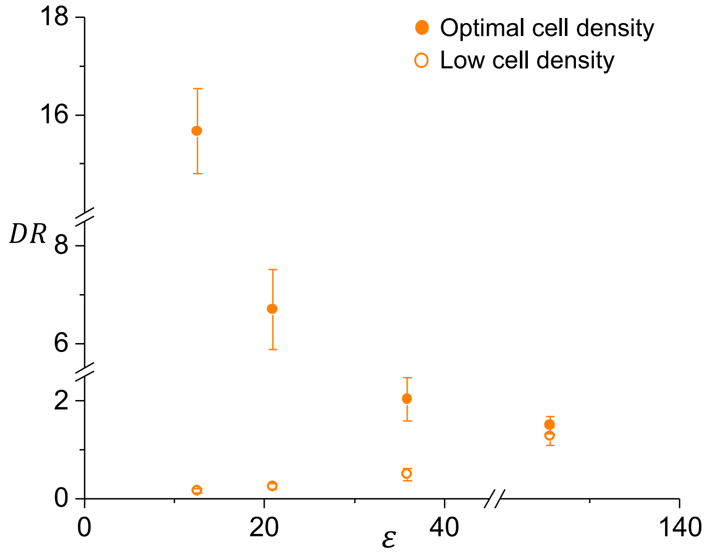

Figure S15: Cells are predominantly found at the center of the soft 2 kPa microwells at low cell density. Plot depicting cell distribution at optimal (solid circles) and low (open circles) cell density in soft 2 kPa microwells in relation to the microwell aspect ratio (ε). The data presented are mean±SD quantified from 100 cells for each aspect ratio and repeated three times

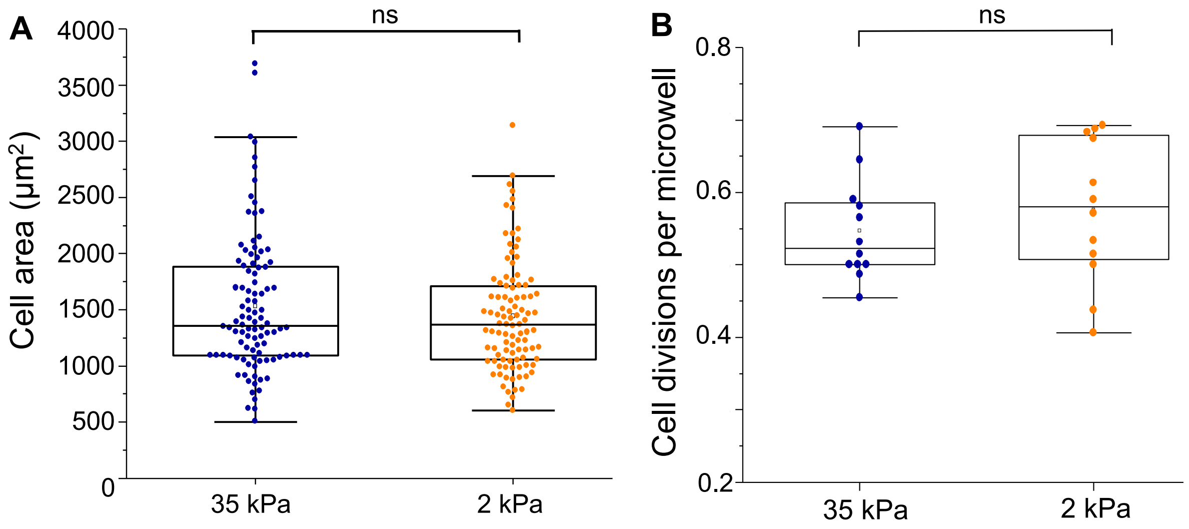

Figure S16: Cell area and proliferation are similar in 35 kPa and 2 kPa microwells. (A) Plot depicting the projected area of cells in 35 kPa and 2 kPa microwells measured by projecting the z-stack on a 2D plane. Data presented are quantified from 100 cells selected from three independent experiments. ns signifies $P > 0.05$ as calculated using Mann-Whitney test. (B) Plot depicting cell divisions occurring in a 35 kPa and 2 kPa microwell in 20 hours. Data presented are collected from twelve time-lapse movies from three independent experiments. ns signifies $P > 0.05$ as calculated using Student's t-test (two-tailed).

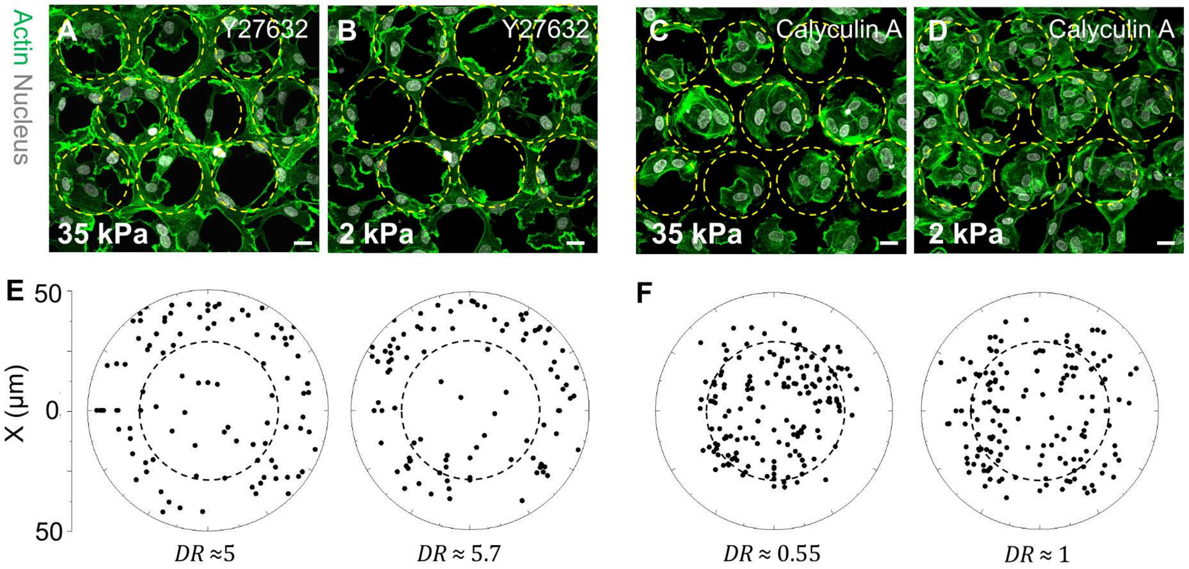

Figure S17: Reduced cell cytoskeleton contractility enhances self-organization. (A, B) Confocal images showing top view of Y27632 treated cells stained for actin (green) and nucleus (gray) in stiff 35 kPa (A), and soft 2 kPa microwells (B). (C, D) Confocal images showing top view of Calyculin A treated cells actin (green) and nucleus (gray) in stiff 35 kPa (C), and soft 2 kPa microwells (D). Scale bar = 25 µm. Yellow dashed circles depict the microwell location. (E,F) Polar plot depicting the distribution of cells in the microwell corresponding to conditions in (A, B, C, and D). Data presented are from 100 cells in each condition pooled from three independent experiments.

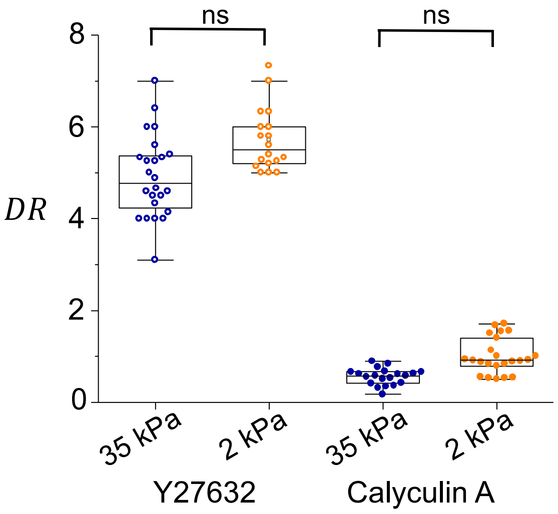

Figure S18: Plot depicting the distribution ratio (*DR*) for cells treated with Y27632 (reduced cell contractility) or Calyculin A (increased cell contractility) in 35 kPa and 2 kPa microwells. Data presented are collected from >15 sample regions from three independent experiments. ns signifies $P >$ 0.05 as calculated using Student's t-test (two-tailed).

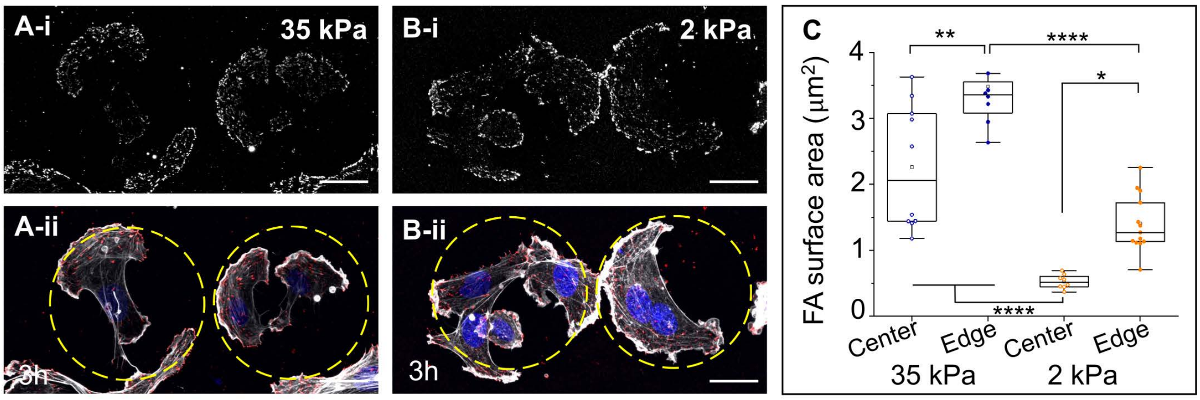

Figure S19: FA distribution in microwells 3 h after seeding. (A, B) Confocal images showing the top view of cells immunostained for paxillin (gray) 3 h after seeding in stiff 35 kPa (A-i) and soft 2 kPa (B-i) microwells and the corresponding composite top view of cells stained for paxillin (red), actin (gray) and nucleus (blue) (A, B-ii). Yellow dotted circles indicate the microwell position. Scale bar = 25 µm. (C) Box-whisker plot depicting paxillin containing FA surface area at the center and edge of stiff 35 kPa and soft 2 kPa microwells. Data presented are for n ≥8 cells pooled from 3 independent experiments. *P=0.027, **P=0.018, and ****P <0.0001 calculated by one-way ANOVA followed by Tukey's mean comparison.

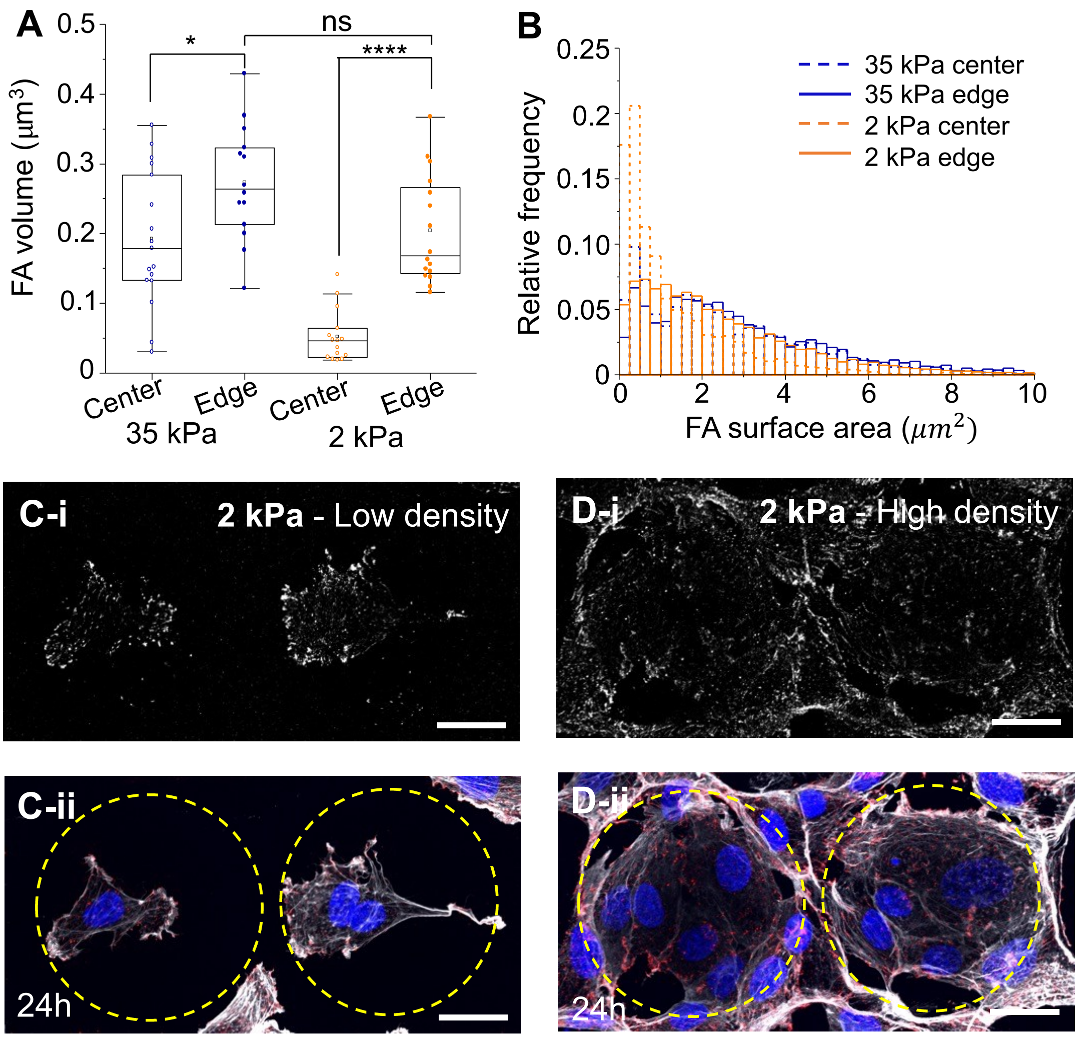

Figure S20: FA distribution in microwells 24 h after seeding. (A) Box-whisker plot depicting FA volume at the center and edge of stiff 35 kPa and soft 2 kPa microwells. Data presented are for n ≥15 cells from three experiments. ns signifies $P > 0.05$, $*P = 0.028$ and $****P < 0.0001$ calculated from one-way ANOVA followed by Tukey's mean comparison. (B) Histogram depicting the relative frequency of FA of a particular size. (C, D) Confocal images showing the top view of cells in soft 2 kPa microwells immunostained for paxillin 24 h after seeding at low density (C-i) and high density (D-i) and the corresponding top view of cells stained for paxillin (red), actin (gray), and nucleus (blue) (C, D-ii). Yellow dotted circles indicate the microwell position. Scale bar = 25 µm.

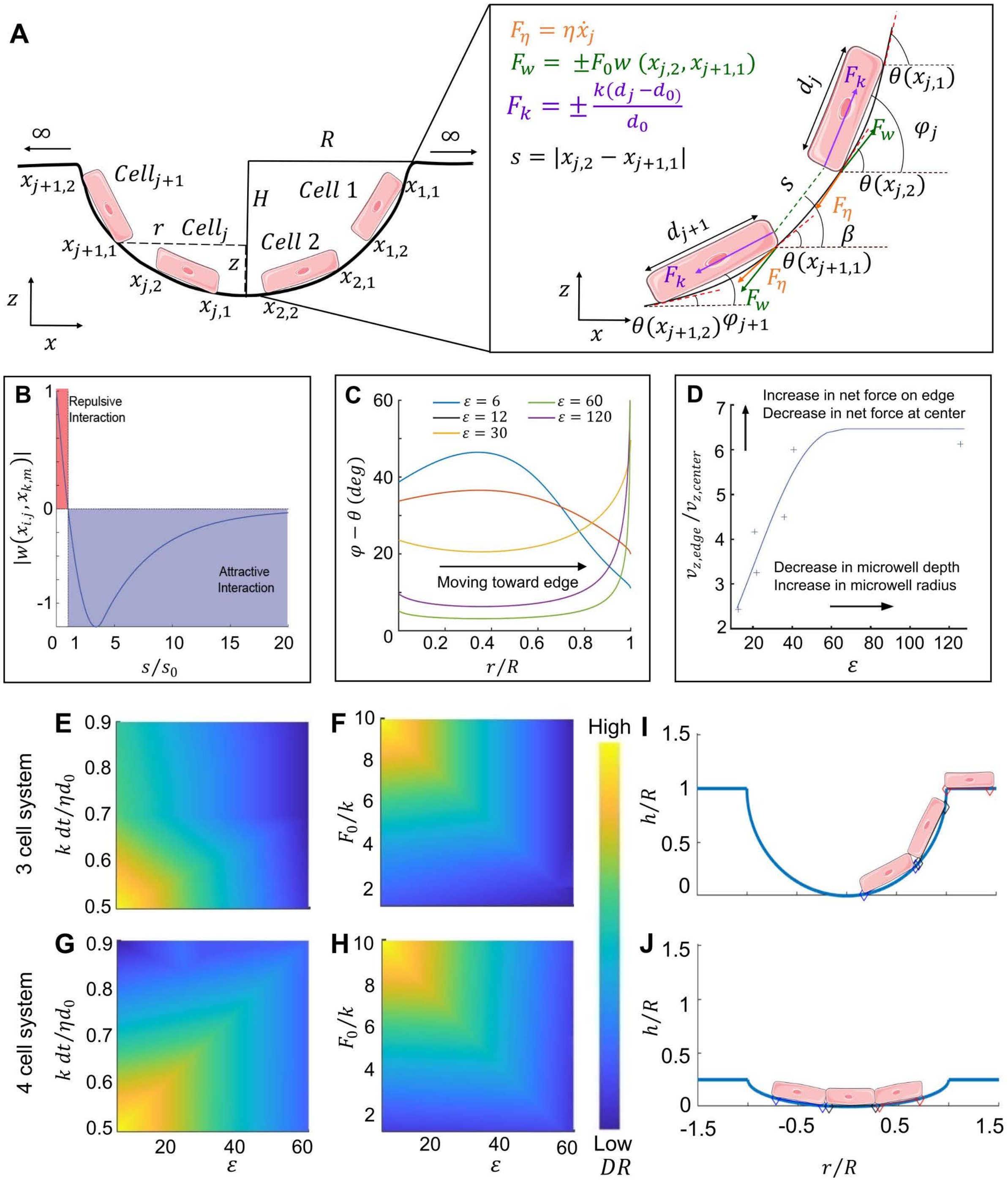

Figure S21: Force balance model predicts multicellular organization. (A) Schematic depicting the force balance model used to simulate the cell organization. (B) Cell-cell attractions along the linear direction modeled using the van der Waals relation. (C) Orientation of the cell relative to the microwell tangential direction at different microwell locations for various aspect ratios. (D) Plot depicting the vertical frictional force as a function of microwell aspect ratio (ε). (E-H) Simulation results for 3 (E, F) and 4 (G, H) cell system. Plots (E,G) show the dependence of *DR* on cytoskeletal tension (related to microwell stiffness) in microwells with a varying ε. Lower ε microwells containing cells with lower cytoskeletal tension have a high *DR* which decreases as tension or ε increases. Plots (F,H) show the effect of increasing cell-cell interactions (related to cell density) in microwells of different ε on *DR*. For a particular ε, the *DR* decreases as cell-cell interactions reduce (or cell density decreases below optimal cell density). (I-J) Steady-state configurations of 3-cell model in case of a deeper (I) and a shallower (J) microwell. In deeper microwells, cells tend to move towards the edge, whereas in the case of shallower microwells, the cells stay at the center.

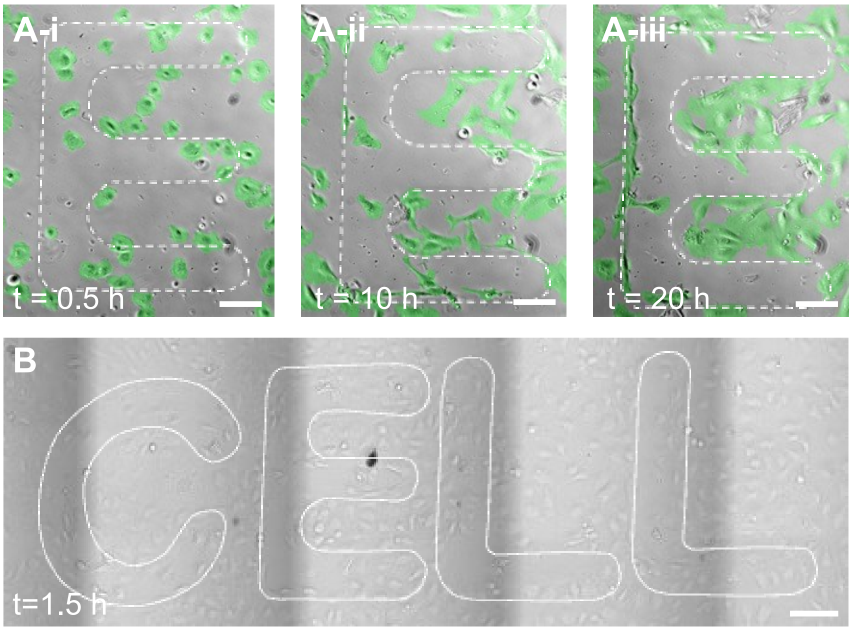

Figure S22: Cell organization on soft hydrogels micropatterned with the word CELL. (A i-iii) False colored (green) time-lapse sequence of cells moving in the letter E. (B) Bright-field image showing cells on the pattern CELL 1.5 h after cell seeding. Scale bar = 100 µm. The white lines indicate the outline of the microwell alphabet patterns.

Table S1: Photoresist type and thickness used to make the silicon master mold for different microwell dimensions. After patterning the photoresist using a CAD mask, the photoresist patterns were heated to 150 °C to induce a curved profile.

Microwell Perimeter (µm)	Aspect ratio	Photoresist height coated (µm)	Photoresist
250	~10	20	AZ 9260 at 1000 rpm
250	~21	10	AZ 9260 at 2000 rpm
250	~36	5	SPR 220 at 3000 rpm
250	~125	1.5	AZ 5214E at 3000 rpm
470	~7.8	50	AZ9260 double coat at 800 rpm
470	~15.7	25	AZ 9260 at 1000 rpm + AZ 9260 at 2000 rpm
470	~31	10	AZ 9260 at 2000 rpm
470	~67.2	5	SPR 220 at 3000 rpm
470	~235	1.5	AZ 5214E at 2000 rpm

Table S2: Cell division events in stiff 35 kPa and soft 2 kPa microwells per 20 h determined from time-lapse imaging.

E	Cell divisions	Number of microwells	Cell divisions/microwell	E	Cell divisions	Number of microwells	Cell divisions/microwell
35 kPa	17	34	0.50	2 kPa	19	31	0.61
	19	39	0.49		22	32	0.69
	15	33	0.45		16	32	0.50
	18	35	0.51		16	30	0.53
	17	32	0.53		20	35	0.57
	23	39	0.59		14	32	0.44
	25	43	0.58		23	39	0.59
	29	45	0.64		13	32	0.41
	26	46	0.57		27	39	0.69
	19	38	0.50		29	43	0.67
	29	42	0.69		28	41	0.68
	20	40	0.50		18	35	0.51

Table S3: Description of parameters used in the force-balance model.

Parameters	Description	Value/Range
$\varepsilon = \frac{2\pi R}{H}$	Ratio between microwell perimeter and depth	$2\pi \times (1\sim20)$
$\frac{d_0}{R}$	Scaled equilibrium length of the cell	0.5
$\frac{s_0}{d_0}$	Scaled equilibrium distance of van der Waals potential	0.1
σ	Standard deviation of random protrusions/contractions distribution	1
$\frac{kdt}{\eta d_0}$	Scaled spring constant	0.5~09
$\frac{F_0 dt}{\eta d_0}$	Scaled cell-cell interaction strength	$\frac{(1\sim10)kdt}{\eta d_0}$
$\frac{Adt}{\eta d_0}$	Scaled random protrusions/contractions	1
$dt\ (s)$	Time increment	0.01

Movie S1: Motion of HUVECs in stiff 35 kPa and soft 2 kPa microwells. A time-lapse video showing the motion of HUVECs in stiff 35 kPa and soft 2 kPa microwells demonstrating the self-organization of cells on the microwell periphery (edge) only on soft 2 kPa hydrogels. Scale bar = 100 μm.

Movie S2: Motion of HUVECs at very low density in stiff 35 kPa and soft 2 kPa microwells. A time-lapse video showing the motion of independently moving HUVECs (seeded at very low density) in stiff 35 kPa and soft 2 kPa microwells demonstrating that individual cells cannot migrate to the microwell edge and continue to move in the microwell in which they were seeded. However, when cells in soft 2 kPa microwell contact other cells (at timestamp 13 h), they tend to move to the edge with their neighbor. Scale bar = 50 μm.

Movie S3: Self-organization of HUVECs at the edges of a microwell shaped as letter E. A time-lapse video showing the self-organization of HUVECs at the edges of a microwell shaped like the letter E. Scale bar = 100 μm.
